# Testicular Germ Cell Tumors Acquire Cisplatin Resistance by Rebalancing the Usage of DNA Repair Pathways

**DOI:** 10.3390/cancers13040787

**Published:** 2021-02-13

**Authors:** Cinzia Caggiano, Francesca Cavallo, Teresa Giannattasio, Gioia Cappelletti, Pellegrino Rossi, Paola Grimaldi, Darren R. Feldman, Maria Jasin, Marco Barchi

**Affiliations:** 1 Department of Biomedicine and Prevention, University of Rome Tor Vergata, 00133 Rome, Italy; cinzia.caggiano@virgilio.it (C.C.); fra.cavallo@gmail.com (F.C.); teresagiannattasio20@gmail.com (T.G.); gioia.cappelletti10@gmail.com (G.C.); pellegrino.rossi@med.uniroma2.it (P.R.); p.grimaldi@med.uniroma2.it (P.G.); 2 Developmental Biology Program, Memorial Sloan-Kettering Cancer Center, New York, NY 10065, USA; m-jasin@ski.mskcc.org; 3 Department of Medicine, Memorial Sloan-Kettering Cancer Center, New York, NY 10065, USA; feldman@mskcc.org

**Keywords:** TGCT, 53BP1, DNA-PKcs, cisplatin resistance, HR, NHEJ, olaparib (AZD2281)

## Abstract

**Simple Summary:**

Germ cell tumors are a model of curable solid tumors due to their unique sensitivity to cisplatin-based chemotherapy. Patients are typically young adults, and despite high cure rate, about 20% of them do not achieve remission or relapse, and 50% of them succumb to the disease. The mechanisms behind their resistance to therapy are largely unknown. By using Testicular Germ Cell Tumor (TGCT) cell lines as a model, we investigated the mechanism of acquired resistance to cisplatin. We demonstrated that resistance occurred by a fine modulation of the DNA repair pathway choice. Namely, in resistant cells, repair of double-strand breaks by non-homologous end joining was dampened by the reduced expression of TP53-binding protein 1 (53BP1) and DNA-dependent protein kinase (DNA-PKcs). However, cisplatin-induced damage was repaired efficiently by homologous recombination. Additionally, we demonstrate that pharmacological inhibition of poly (ADP-ribose) polymerase (PARP) combined with cisplatin had an additive/synergistic effect on cisplatin-resistant cells, which represents the proof of concept for introducing PARP inhibitors in salvage therapy.

**Abstract:**

Despite germ cell tumors (GCTs) responding to cisplatin-based chemotherapy at a high rate, a subset of patients does not respond to treatment and have significantly worse prognosis. The biological mechanisms underlying the resistance remain unknown. In this study, by using two TGCT cell lines that have acquired cisplatin resistance after chronic exposure to the drug, we identified some key proteins and mechanisms of acquired resistance. We show that cisplatin-resistant cell lines had a non-homologous end-joining (NHEJ)-less phenotype. This correlated with a reduced basal expression of TP53-binding protein 1 (53BP1) and DNA-dependent protein kinase (DNA-PKcs) proteins and reduced formation of 53BP1 foci after cisplatin treatment. Consistent with these observations, modulation of 53BP1 protein expression altered the cell line’s resistance to cisplatin, and inhibition of DNA-PKcs activity antagonized cisplatin cytotoxicity. Dampening of NHEJ was accompanied by a functional increase in the repair of DNA double-strand breaks (DSBs) by the homologous recombination repair pathway. As a result, cisplatin-resistant cells were more resistant to PARP inhibitor (PARPi) monotherapy. Moreover, when PARPi was given in combination with cisplatin, it exerted an additive/synergistic effect, and reduced the cisplatin dose for cytotoxicity. These results suggest that treatment of cisplatin-refractory patients may benefit from low-dose cisplatin therapy combined with PARPi.

## 1. Introduction

Since the mid-1970s, when cisplatin-based therapy was first introduced, testicular germ cell tumors (GCTs) have been considered a model of curable solid tumors. However, approximately 20% of all patients are not cured with standard first-line chemotherapy. Furthermore, only 50% of patients progressing after first-line chemotherapy can be cured with salvage treatment, with the rest destined to succumb to the disease [1]. Since these tumors typically occur in 15- to 44-year-old men [2], an average of nearly 40 years of life is lost when a patient dies from testicular cancer [3]. Hence, there is an urgent clinical need to determine the mechanisms behind cisplatin resistance and to identify new targeted therapies able to increase the survival rate of these young men.

Based on histology, testicular GCTs can be classified as seminomas and non-seminomas. The latter can be further classified as teratoma, yolk sac tumor, choriocarcinoma, embryonal carcinoma (EC) and mixtures of two or more components—the mixed tumors [4]. ECs are considered the malignant stem cell component of non-seminomas; their presence, along with concomitant vascular invasion, represents a risk factor for metastasis or recurrence in early-stage disease [5,6,7,8]. Testicular GCTs, in particular ECs, are intrinsically hypersensitive to drug-induced cell death [9]. However, the mechanisms behind this exquisite cisplatin sensitivity, as well as those accounting for chemotherapy resistance, remain poorly understood.

Cisplatin causes multiple types of DNA damage, among them interstrand crosslinks (ICLs). Despite ICLs representing only a small fraction of cisplatin-induced damage [10], they are considered the most cytotoxic and genotoxic lesions caused by the drug. Indeed, ICLs covalently link the two strands of the double helix, creating a roadblock to replication and transcription [11]. Removal of ICLs in the S phase of the cell cycle requires, in mammalian cells, the orchestration of multiple DNA repair processes that encompass recognition of the ICL, removal of the adduct by DNA incision with formation of DNA double-strand breaks (DSBs) through the activation of the Fanconi Anemia (FA) pathway, unhooking of the ICL adduct, DNA synthesis and repair of the DSBs by homologous recombination (HR). Given the importance of ICLs in cisplatin-induced cytotoxicity, it is likely that the exquisite sensitivity of testicular GCTs to cisplatin results, at least in part, from impairment of one or more steps of the ICL repair mechanism. Furthermore, this intrinsic property of GCTs may represent a potential target for treatment of tumors which acquire cisplatin resistance. Accordingly, we have previously demonstrated that EC cell lines have a relatively low proficiency in HR and that their sensitivity to cisplatin parallels HR deficiency [11]. However, whether HR proficiency is increased in testicular tumors that are refractory to cisplatin-based chemotherapy remains unknown, as well as the possible involvement of additional repair mechanisms [12].

HR is a conservative repair mechanism of DSBs that operates in the S/G2 phase of the cell cycle using the sister chromatid as a repair template. Breast cancer type 1 susceptibility protein (BRCA1) and C-terminal binding protein-1 interacting protein (CtIP) are key factors promoting the first step of HR, DSB resection. This leads to the formation of single-strand DNA (ssDNA) intermediates that are the docking sites of Replication Protein A (RPA) and DNA repair protein RAD51 homolog 1 (RAD51), two pro-HR factors that promote the strand invasion step that characterizes HR [13]. In addition to HR, DSBs are also repaired in mammalian cells by non-homologous end joining (NHEJ). The latter operates throughout the cell cycle and mediates the direct re-ligation of the DSBs’ end in an error-prone fashion [14]. HR and NHEJ compete for the repair of DSBs during replication, and repair pathway choice relies on the balance of pro-HR and pro-NHEJ factors at DSB sites. Key pro-NHEJ factors are the DNA damage response protein TP53-binding protein 1 (53BP1), ATP-dependent DNA helicase 2 subunit KU70/KU 80 (KU70/KU80) and DNA-dependent protein kinase (DNA-PKcs). By assembling at DSB-flanking chromatin, 53BP1 inhibits extensive resection of DNA ends, steering repair away from HR [15,16,17], while KU70/KU80 and DNA-PKcs are involved in later steps of blunt end ligation of DSBs [14]. NHEJ is dispensable for DSB repair after ICL incision [18]. However, preventing the utilization of NHEJ in ICL repair-deficient cells has been reported to substantially rescue their sensitivity to cisplatin [19,20] and also increases survival of wild-type (WT) cells treated with the drug [20,21]. This suggests that proper usage of NHEJ contributes to promote cisplatin resistance, although a recent study has called this into question [22].

In this study, we demonstrate that EC cells that acquire cisplatin resistance following chronic cisplatin exposure have reduced 53BP1 and DNA-PKcs protein expression and that such reduction is critical to their acquired resistance to cisplatin. Moreover, we show that dampening of NHEJ in cisplatin-resistant (cis-r) cells parallels an increase in the proficiency in HR. Therefore, cis-r cells are resistant to poly (ADP-ribose) polymerase inhibitor (PARPi) monotherapy, a drug that targets HR-deficient tumors. Moreover, we show that PARPi interacts synergistically with cisplatin, indicating the potential for its use in cisplatin-refractory patients.

## 2. Materials and Methods

### 2.1. Cell Lines and Culture

GCT27 is an EC cell line established from a previously untreated patient whose cell biological properties have been reported [23]. GCT27cis-r cells were obtained by exposure to chronic increasing concentrations of cisplatin as previously described [24]. Paired cell lines were kindly provided by Francesc Viñals (Catalan Institute of Oncology, Universitat de Barcelona-IDIBELL, Spain). The cell lines were authenticated in 2012 by the Authentication service of Health Protection of Agency Culture, United Kingdom [25]. The cell line 2102EP is an EC cell line derived from a primary human testicular teratocarcinoma [26]. The 2102EPcis-r subline was created by long-term culture of 2102EP cells, under increasing concentrations of cisplatin [27]. Paired 2102EP cell lines were generously provided by Dr. Michael Höpfner (University of Berlin, Berlin, Germany). EC cell lines were cultured in Dulbecco’s high-glucose Modified Eagle Medium (DMEM; Sigma-Aldrich, St. Louis, MO, USA) supplemented with 10% fetal bovine serum (FBS) (GIBCO, Waltham, MA, USA), antibiotics and L-glutamine (GIBCO). EC cell lines were harvested using 0.5X trypsin-EDTA (Aurogene, Rome, Italy). MCF10A cells were cultured in DMEM supplemented with 5% horse serum, antibiotics, epidermal growth factor (EGF) (100 µg/mL, Sigma-Aldrich), hydrocortisone (1 mg/mL, Sigma-Aldrich), cholera toxin (1 mg/mL, Sigma-Aldrich) and insulin (10 mg/mL, Sigma-Aldrich). MCF10A cells were harvested using 1X trypsin-EDTA (Aurogene). All cells were grown at 37 °C in a humified atmosphere with 5% CO_2_. During this study, relative resistance of all cell lines to cisplatin was periodically checked and remained stable in the absence of further maintenance doses of cisplatin. All cells were periodically checked for and found to be free of *Mycoplasma*.

### 2.2. Drugs

Cisplatin (Sigma-Aldrich 479306) was dissolved in sterile 0.9% NaCl to a 3-mM concentration and stored at −80 °C until use, avoiding thawing/freezing cycles. Etoposide (Cayman Chemical Company, Ann Arbor, MI, USA; n. 12092) was dissolved in dimethyl sulfoxide (DMSO) to 50 mM and stored in aliquots at −20 °C. Bleomycin sulfate (Molekula Group, Darlington, UK; n. 25097489) was dissolved in sterile 0.9% NaCl to a 0.5-mM concentration and stored in aliquots at −80 °C. NU7441 (Cayman chemical company n. 14881) was dissolved in DMSO to a 5-mM concentration and stored in aliquots at −20 °C. The PARPi AZD2281 was provided as powder by the Memorial Sloan-Kettering Cancer Center and dissolved in DMSO to a concentration range of 10^−8^ to 10^−4^ M and stored in aliquots at −20 °C. Cytochalasin B (Cayman chemical company; 11328) was dissolved in ethanol to a 50-mM concentration and stored in aliquots at −20 °C.

### 2.3. Micronuclei (MN) Assay

The day before treatment, EC cells were seeded on glass slides in 24-well culture plates at a density of 10–15 × 10^3^ cells/well. After an overnight (ON) culture, cells were exposed to 3 μM cisplatin for 6 h, washed out twice with Phosphate-Buffered Saline (PBS) (ECB4004L Euroclone, Pero, Italy) and cultured in drug-free media for 12 h. At the end of the incubation, cells were exposed to 5 μM cytochalasin B for 24 h to inhibit cytoplasmic division. After treatment, cells were washed twice with ice-cold PBS and fixed with cold methanol at −20 °C for 15 min. Cells were than washed with ice-cold PBS and permeabilized in 0.5× triton X-100 + 0.5% goat serum in PBS for 15 min at room temperature (RT) and were next blocked in 0.2% goat serum in PBS for 1 h at RT. After two washes with PBS, cells were incubated successively with the anti-tubulin antibody (1:5000; Sigma-Aldrich; n. T4026) for 2 h at RT and with the goat anti-mouse antibody Alexa-488 for 1 h at RT. DNA and micronuclei (MN) were stained with Hoechst 33342. MN were counted only in binucleated cells, using a Leica DMI 6000B microscope equipped with a DFC 350 FX camera (Leica, Wetzlar, Germany).

### 2.4. Colony Forming Assay

Cells were seeded at known low densities into either a 10-cm plate (2000–3000 cells/plate) or a 6-well plate (100–300 cells/well) in three replicates per condition. After 18 h, the cell culture media were substituted with fresh media, either without (untreated control) or containing drugs (either cisplatin, etoposide, bleomycin, AZD2281 or a combination of cisplatin with AZD2281) at the appropriate concentrations. Cells were than incubated with the drugs for 6 h to 24 h accordingly with the administration schedule. At the end of treatment, drugs were removed, and after two washouts with 1× PBS, they were cultured in the presence of AZD2281 at the appropriate concentration. Treatment was performed for 6 h or extended up to 7–10 days (6-well plate) or to 14 days (10-cm plates) accordingly with the treatment schedule. In the long-term exposure experiments with AZD2281, the drug was renewed every 24 h and cells were grown until colonies containing at least 50 cells/clone were visible in untreated controls. At the end of the incubation time, the culture media were removed, and colonies were dried out ON under a chemical hood. Colonies were quantified by staining with 2% methylene blue, 1% crystal violet in H_2_O for 3–4 h at room temperature (RT). At the end of the incubation, the excess of the staining solution was removed, and after a wash with ddH_2_O, colonies were left to dry out under a chemical hood for 2–3 h before quantification of colony number. In all experiments, data were normalized to the untreated conditions to consider variations in plating efficiency.

### 2.5. Cell Cycle Analysis and Quantification of Phospho-H2AX (Ser139) Signal by Flow Cytometry

Approximately 500,000 cisplatin-treated and untreated cells were collected, fixed in cold 70% ethanol and stored at −20 °C for up to 1 week before analysis. Cells were washed in Tris-buffered saline (TBS), pH 7.4, and rehydrated for 10 min at RT in TBS containing 4% (Bovine Serum Albumin) BSA and 0.1% Triton X-100 (TST) (Sigma-Aldrich). Cells were stained with anti-phospho-Histone H2AX (Ser139) monoclonal antibody (mAb) (Upstate Biotechnology, New York, NY, USA) diluted at 1:250 in TST for 2 h at RT. After two washes with TBS, cells were incubated with secondary antibody diluted at 1:200 (Fluorescein isothiocyanate (FITC)-conjugated Invitrogen Alexa Fluor 488) in TST for 1 h at RT. Next, cells were resuspended with 50 μg/mL RNase A and 100 μg/mL propidium iodide (PI; Sigma-Aldrich) and incubated for 1 h at 37 °C. A minimum of 10,000 stained cells were acquired on a FACScan flow cytometer (Becton Dickinson, Franklin Lakes, NJ, USA) and analyzed with the Flowjo software.

### 2.6. Immunodetection of DNA Damage Repair Foci

TGCT cell lines were plated on a Nunc Lab-Tek chamber slide and, 16 h later, were exposed to 3 μM cisplatin for 6 h or left untreated. To detect cells in the S phase of the cell cycle, 30 min before the staining, cells were incubated with 30 μM (5-Bromo-2′-Deoxyuridine (BrdU) (Sigma-Aldrich). At 6 h following exposure and 16 h after cisplatin washout, cells were fixed with fresh 4% paraformaldehyde, washed with PBS, incubated with 1 M HCl for 20 min, washed with PBS and incubated in 0.1 M sodium tetraborate, pH 8.5, for 2 min at RT and washed again with PBS. Cells were permeabilized with PBS 0.5% Triton X-100 plus 0.5% normal goat serum (NGS) at RT, then incubated in PBS 0.2% Triton X-100 and blocked with 0.2% NGS in PBS at RT for 1 h. To identify cells in the S/G2 phase of the cell cycle, cells were stained by the anti-cyclin A antibody (1:250, Santa Cruz Biotechnology, Dallas, TX, USA; H-432). Cells in the S phase of the cell cycle were identified by adding BrdU to the culture media 30 min before stopping the culture. BrdU was detected using a mouse anti-BrdU antibody (1:200; Pharmigen, Franklin Lakes, NJ, USA; 33281A). Other primary antibodies used were as follows: anti-H2AX Ser139 (1:250; Cell Signaling 978), anti-RPA1 (1:250; Santa Cruz Biotechnology B6), anti-RAD51 (1:250 H-92; Santa Cruz Biotechnology), anti-BRCA1 (1:100 D9; Santa Cruz Biotechnology), anti-FANCD2 (1:250; Novus Biologicals, Centennial, CO, USA; NB100-182) and anti-53BP1 (1:250; OriGene, Rockville, MD, USA; ta309918). Primary antibodies were either incubated for 90 min at RT or ON at 4 °C. Secondary antibodies were goat anti-mouse antibody Alexa-488 or goat anti-rabbit Alexa-594 (1:1000; Invitrogen, Carlsbad, CA, USA). Nuclei were scored using a Leica DMI 6000B microscope equipped with a DFC 350 FX camera (Leica).

### 2.7. DNA Repair Assays

DSB repair was measured by using the Dr-GFP and Tr-GFP assays as described previously [28]. The measure of DSB repair by NHEJ was performed using the EJ5-GFP reporter substrate as described [29], with minor modifications. In brief, following ON culture, subconfluent 10-cm plates of EC cells were trypsinized with trypsin citrate [28], washed once with PBS and resuspended to determine cell density. Then, 2 × 10^6^ cells were resuspended in 100 μL of the Nucleofactor^TM^ L buffer (Amaxa, Lonza Group, Basel, Switzerland) and electroporated with 1 μg pEJ5-GFP and 3 μg pCBA*Sce*I or pCAGGS (used as a negative control) using the Nucleofactor device (Amaxa) employing the program A-20 [28]. The percentage of GFP+ cells was measured by Flow Cytometry (FACS), 48 h upon transfection for Dr-GFP and NHEJ assays, or 48 h upon UV irradiation for the Tr-GFP assay. The GFP-expressing vector pNZE-GFP was used to measure transfection efficiency in side-by-side experiments. GFP+ cells were quantified by FACS. In all cases, the repair efficiency was normalized against transfection efficiency.

### 2.8. Crystal Violet Assay

Cells were seeded in a 96-well plate at the appropriate density (2102EP 3.5 × 10^3^ cells/well; GCT27 2.5 × 10^3^ cells/well) and, the next day, were incubated with the appropriate drugs, accordingly with the experimental setting. At the end of the incubation time, cells were washed with PBS and dried out under a fume hood for 2–3 h. Then, 100 μL of crystal violet solution (0.2% in ddH_2_O) was added in each well and washed out with ddH_2_O after 30 min incubation. Plates were then left to dry out under a fume hood and the optical density (OD) of each well was measured using a plate reader (OD570 nm). The OD of the untreated control cells was set to 100%, and stimulated samples were normalized against the control.

### 2.9. Generation of Stable Cell Lines

To stably increase the expression of 53BP1 in 2102EPcis-r cells, we infected them using retroviral particles expressing either the N-Myc-53BP1 WT pPLC-Puro vector (Addgene, Watertown, NY, USA, 19836) or pLPC-N MYC (Addgene, 12540) as a control. Overexpressing cells were selected by culturing them in presence of 1 μg/mL puromycin until single colonies were visible. Overexpression of 53BP1 in single clones was monitored by Western blotting by comparing it side-by-side to the expression in 2102EPcis-s cells and 2102EPcis-r cells infected with the control vector. Silencing of 53BP1 in GCT27cis-s cells was obtained by lentiviral infection with particles containing either the pGIPZ lentiviral 53bp1 shRNAmir-1 (Dharmacon, Lafayette, LA, USA clone ID: V3LHS_635699; sequence: TGACAGTTGAGTGTTCTAA) or pGIPZ lentiviral 53bp1 shRNAmir-2 (Dharmacon clone ID: V3LHS_635694; sequence: TGAGTCAGAATGATGACAA) or a non-silencing control vector.

### 2.10. Immunoblotting and Biochemical Fractionation

Whole-cell extract preparation was performed using lysis buffer (50 mM Hepes, pH 7.4, 150 mM NaCl, 15 mM MgCl_2_, 10% glycerol, phosphatase and protease inhibitor cocktails (Roche, Basel, Switzerland), 0.5% Triton X-100). After 10 min incubation on ice, the cell suspension was centrifuged for 10 min at 12,000× *g* at 4 °C, and the supernatant fractions were collected and used for further analyses.

Cell fractionation was obtained through two consecutive extractions. First, nuclei isolation was performed using ice-cold nuclei EZ lysis Buffer (Sigma Aldrich) according to the manufacturer’s instructions. Second, chromatin-bound protein fraction was isolated by diluting the nuclei pellet in ice-cold extraction buffer (50 mM Hepes, pH 7.5, 150 mM NaCl, 1 mM EDTA supplemented with phosphatase and protease inhibitor cocktails) containing 200 μg/mL RNase A and incubating for 30 min at 25 °C under agitation. Following centrifugation at 14,000× *g* for 3 min, the pellet was resuspended in PBS buffer supplemented with 1% SDS, heated for 10 min at 100 °C and sonicated for 10 s. Concentrated loading sample buffer was added for a 1× final concentration in all protein lysates, and the samples were boiled for 5 min. Western blot analysis was carried using the antibodies listed in Table 1.

### 2.11. Determination of Synergy

The interaction between AZD2281 and cisplatin was characterized using a non-constant drug ratio and was analyzed with the combination index (CI) method using the CompuSyn and/or CalcuSyn software.

### 2.12. In Silico Analysis of Progression-Free Survival

To investigate the correlation between *TP53BP1* and *PRKDC* genes expression with tumor recurrence, we retrieved a public dataset (TCGA, PanCancer Atlas) from cBioPortal (https://www.cbioportal.org/, accessed on 29 December 2020) and performed the Kaplan–Meier Plot using a free survival analysis tool (http://kmplot.com/analysis/index.php?p=service, accessed on 29 December 2020) [30]. We included, in the analyses, the mRNA expression z-scores relative to all samples’ (log RNA Seq V2 RSEM) values of the following non-seminoma subtypes: ECs, teratomas and mixed TGCTs with EC components. A total of 51 patients were analyzed. Cut-off median values used in the analyses were −0.96 for *TP53BP1* and 0.15 for *PRKDC*.

### 2.13. Statistical Analysis

All experimental results were analyzed using an unpaired one- or two-tailed Student’s *t*-test (*p* < 0.05) as indicated in figure legends, using Prism 8 software.

## 3. Results

### 3.1. Testicular GCT Cell Lines with Acquired Resistance to Cisplatin Show Improved DNA Damage Repair Proficiency and Decreased Genome Instability

In this study, we made use of two cis-r cell lines: GCT27cis-r and 2102EPcis-r, obtained by chronic exposure of their cis-s parental cell lines to increasing concentrations of cisplatin [24,27]. To confirm the acquired resistance, we performed a clonogenic colony formation assay (hereafter named colony assay) after a 6 h pulse of 3 μM of cisplatin (corresponding to the median plasma concentration measured in testicular tumor patients treated with cisplatin [31]). We found that the GCT27cis-r and 2102EPcis-r cell lines were more resistant to cisplatin (Figure S1A,B), with a resistance factor (RF) determined from the ratio of half-maximal inhibitor concentrations (IC_50_ cis-r/IC_50_ cis-s) of 4 and 3.6, respectively, and an RF determined from IC_90_ ratios increased by over fivefold (Table 2).

In standard first-line chemotherapy regimens, cisplatin is given in combination with two other DSB-inducing agents: etoposide and bleomycin [1]. To understand whether cis-r cells were also co-resistant to the above drugs, we tested their sensitivity using a colony assay. We observed that both cis-r cell lines were co-resistant to the drugs (Figure S1C–F).

**Figure 1:**
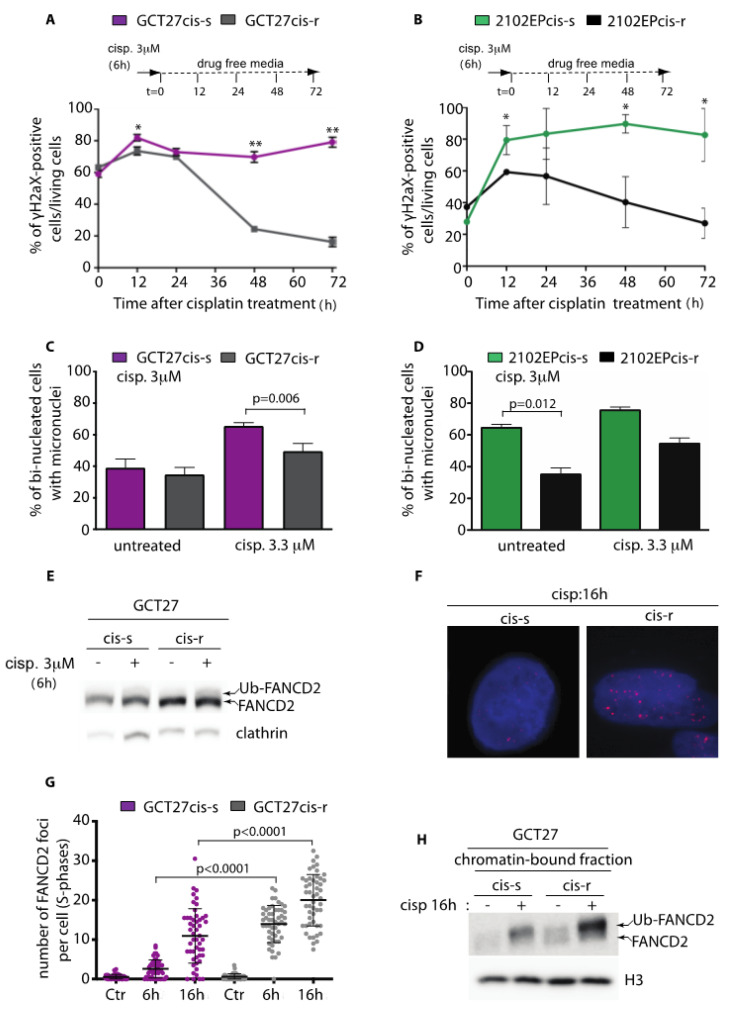
(**A**,**B**) Flow cytometry of γH2AX-positive cells upon cisplatin treatment. Embryonal carcinoma (EC) cell lines were treated with a pulse of cisplatin for 6 h and collected for analysis at the indicated time points after drug washout. Data are mean values ± s.d. of three independent experiments. (**C**,**D**) Percentage of binucleated cells with one or more micronuclei in untreated and cisplatin-treated cells. Data are mean values ± s.d. of two independent experiments. (**E**) Western blotting analyses of FANCD2 expression in GCT27 paired cell lines before and after treatment with 3 μM cisplatin for 6 h. Arrows point to the active monoubiquitinated (Ub-FANCD2) and inactive non-monoubiquitinated (FANCD2) forms of the protein. Clathrin was used as a loading control. Original blots see Figure S3A (**F**) Immunofluorescence of FANCD2 (red) in GCT27 cells collected at the indicated time point after drug washout. Cells were counterstained with Hoechst (blue) to identify nuclei. (**G**) Quantification of FANCD2 foci in GCT27 paired cell lines after a 6 h pulse with 3 μM cisplatin. Foci were counted in S-phase (BrdU-positive) nuclei, both at the end of treatment (6 h) and after 16 h of culture in drug-free media. Data are mean values ± s.d. of three to four independent experiments. (**H**) FANCD2 Western blotting analysis from chromatin extracts of GCT27 paired cell lines collected before (-) and after (+) treatment with cisplatin. Histone H3 (H3) was used as a loading control. Original blots see Figure S3B Statistical analyses were performed using the unpaired two-tailed *t*-test (* *p* < 0.05; ** *p* < 0.01).

The observation that cis-r cells were co-resistant to multiple DSB-inducing agents suggested that these cells were able to overcome a DSB lesion with greater efficiency. To test this hypothesis, we treated unsynchronized cells with a 6 h pulse of 3 μM of cisplatin and analyzed their DNA damage repair dynamic by staining for H2AX Ser139 (γH2AX), a surrogate marker of DSBs [32]. We observed that while levels of γH2AX were similar in both cis-s and cis-r cells lines upon cisplatin treatment (t = 0 in Figure 1A,B), 12 h after drug washout, the γH2AX signal was significantly higher in cis-s cell lines (likely because of formation of DSBs at the collapsed replication forks [11,33]) and remained high for up to 72 h. On the contrary, γH2AX decreased by 48 h after release in cis-r cells (Figure 1A,B). This suggested that cis-r cells repaired DNA damage with greater efficiency. However, one alternative interpretation could be that the load of DNA damage induced by the treatment in cis-s cells was too high to be repaired with the same kinetics of cis-r cells. To challenge this hypothesis, we treated cis-s and cis-r cells with a dose of cisplatin equivalent to the IC_50_ of each cell line and analyzed their repair kinetics after drug release. Again, cis-s and cis-r cells differed in their response to cisplatin-induced damage. Even though cisplatin induced an identical load of DNA damage (t = 0 in Figure S2A), we observed that repair upon drug release occurred more efficiently in the GCT27cis-r cell line than in the cis-s counterpart (Figure S2A). The analysis of 2102EP paired cell lines revealed that under these experimental conditions, the load of DNA damage induced by cisplatin in 2102EPcis-r was reduced relative to 2102EPcis-s cells (t = 0, Figure S2B). This suggests a slight difference in the proficiency of cell lines in their cisplatin uptake/efflux mechanisms. However, the DNA damage load was comparable among cis-r and cis-s cell lines 12 h after drug washout, and while the damage remained almost unrepaired in 2102EPcis-s cells up to 72 h after release, in the 2102EPcis-r cell line, the γH2AX signal decreased with fast kinetics by 24 h after the drug washout (Figure S2B). In accordance with these results, the percentage of apoptotic cells in the sub-G1 phase of the cell cycle was reduced in both GCT27cis-r and 2102EPcis-r cells, indicating that cells that repaired the damage survived the treatment (Figure S2C,D). These observations confirm that cis-r cells repair cisplatin-induced damage more efficiently.

Micronuclei (MN) are biomarkers of genome instability commonly seen in cancers. MN can originate from missegregation of whole chromosomes at anaphase or from lagging acentric chromosomes or chromatid fragments caused by misrepaired or unrepaired DNA breaks [34]. To analyze if the increased proficiency in repair of cisplatin-induced damage was accompanied by a reduction in genome instability, we counted the number of micronuclei before and after cisplatin treatment in binucleated cells. As shown in Figure 1C,D, even though the basal number of MN differed in untreated cis-r and cis-s cell lines, when paired cell lines were treated with cisplatin, the number of MN counted in cis-r cells was significantly lower than that in cis-s cell lines, indicating a greater stability of the genome. Since genetic stability highly depends on the type of DNA repair pathway utilized [35], we concluded that cis-r cells likely overcome cisplatin-induced damage by employing more conservative mechanisms of recombination, with consequent increased survival after treatment.

### 3.2. GCT27cis-r Cells Activate the FA-Pathway with Higher Efficiency than GCT27cis-s Cells

The FA repair pathway has a key function in genome protection against ICLs. A key event in this pathway is the endonucleolytic incision required for the unhooking of the ICL, carried out by the monoubiquitinated FA complementation Group I-D2 (FANCI-FANCD2) complex [36]. To gain insight on the mechanism of acquired resistance to cisplatin in cis-r cells, we analyzed FANCD2 activation in GCT27 paired cell lines by looking at its monoubiquitination through Western blotting. Unsynchronized GCT27cis-s and GCT27cis-r cells were subjected to a pulse of cisplatin, and total protein extracts were probed with the anti-FANCD2 antibody. In GCT27cis-r cells, basal FANCD2 expression was increased and a greater fraction of total FANCD2 was ubiquitinated after drug treatment (Figure 1E). Ubiquitination of FANCD2 results in its targeting into cytological visible nuclear foci [36]. To confirm FANCD2 hyperactivation, we quantified the number of foci by immunofluorescence, in cells at the S phase of the cell cycle. As shown in Figure 1F,G, we observed that the number of FANCD2 foci was much greater in GCT27cis-r cells than in their cis-s counterparts, both after a 6 h pulse of cisplatin and 16 h after drug washout. The increased binding of Ub-FANCD2 onto DNA was confirmed by probing the chromatin-bound enriched fraction with the anti-FANCD2 antibody (Figure 1H). We concluded that in GCT27cis-r cells, activation of the FA pathway occurs with a higher efficiency than in GCT27cis-s cells. Next, we asked whether a similar mechanism could account for the acquired resistance to cisplatin of 2102EPcis-r cells. As shown in Figure S2E, we observed no increase in FANCD2 foci number in 2102EPcis-r cells after cisplatin treatment. Moreover, beside the FANCD2 basal expression level being increased in 2102EPcis-r cells (Figure S2F), the Ub-FANCD2 subfraction was comparable to that of cis-s cells (Figure S2G). We concluded that in 2102EPcis-r cells, the proficiency of the FANCD2-mediated pathway was comparable to that of 2102EPcis-s cells.

### 3.3. GCT27cis-r and 2102EPcis-r Cells Show an Increased Proficiency of Repair of DSBs by HR

**Figure 2:**
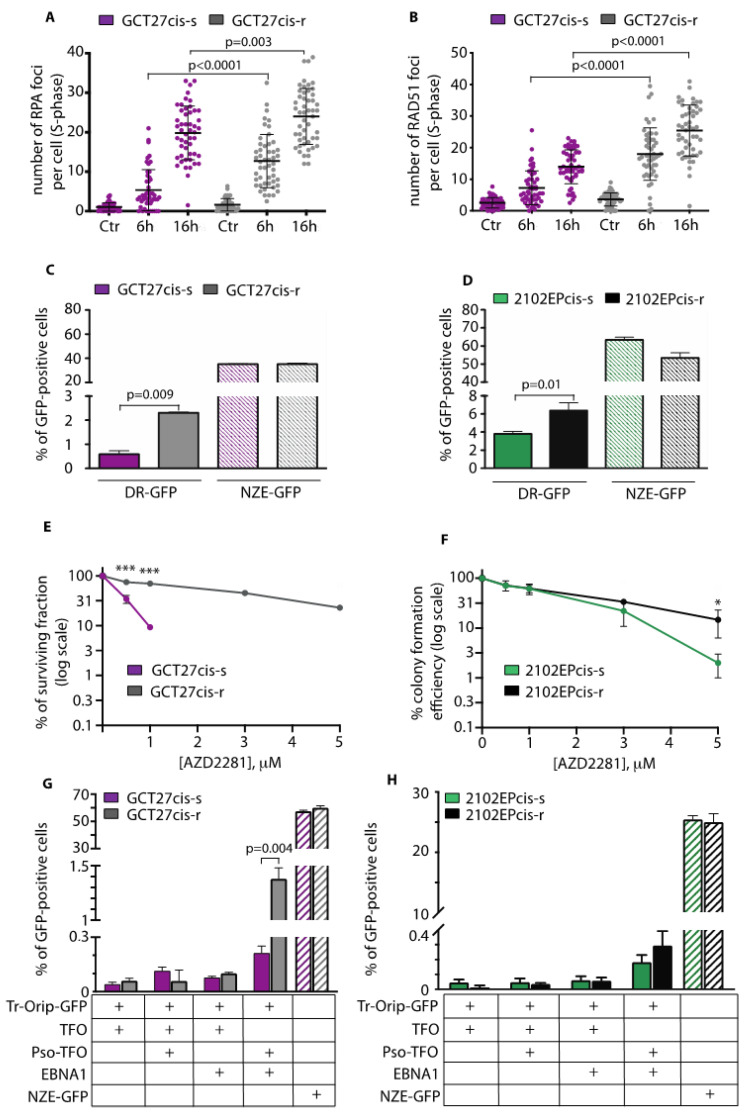
(**A**,**B**) Quantification of RPA and RAD51 foci numbers in BrdU-positive (S-phase) GCT27 paired cell lines, before (Ctr) and after treatment with 3 μM cisplatin (6 h) and 16 h of culture in drug-free media. Data are mean values ± s.d. of three independent experiments. (**C**,**D**) Homology-direct repair proficiency of the indicated cell lines as measured by the DR-GFP assay. NZE-GFP is the plasmid used to measure transfection efficiency of each cell line. Data are mean values ± s.d. of two to three independent experiments. (**E**,**F**) Colony assay of the indicated cell lines upon chronic exposure to increasing doses of AZD2281. Data are mean values ± s.d. of five independent experiments. (**G**,**H**) Homology-direct repair proficiency of the indicated cell lines as measured by the Tr-OriP-GFP assay. Tr-OriP-GFP is the GFP reporter substrate containing an Epstein–Barr nuclear antigen 1 (EBNA1) origin of replication (OriP) and a sequence that can bind a triplex-forming oligonucleotide (TFO) conjugated with psoralen (pso-TFO). NZE-GFP is the plasmid used to measure transfection efficiency. Data are mean values ± s.d. of two independent experiments. In all cases, statistical analyses were *performed* using a two-tailed *t*-test (* *p* < 0.05; *** *p* < 0.0001).

We have previously shown that in EC cell lines, reduced proficiency in HR correlates with sensitivity to cisplatin [11]. To test whether the acquired resistance of GCT27cis-r and 2102EPcis-r cells to cisplatin was linked to an increased proficiency in DSB repair by HR, we quantified the number of RPA and RAD51 foci that assemble in S phase, after treatment with cisplatin. Treated (and untreated) cells were collected both at the end of treatment (6 h) and after 16 h of culture in drug-free media. As quantified in Figure 2A,B, we found that in GCT27cis-r cells, RPA and RAD51 foci were increased significantly compared to their cis-s counterparts at both examined time points. Importantly, such an increase was not due to a difference in cisplatin-induced damage, as documented by the comparable number of γH2AX foci across cell lines (Figure S2H). Similarly, quantification of γH2AX, RPA and RAD51 foci numbers in 2102EPcis-s and 2102EPcis-r cell lines (Figure S2H and S4A,B) revealed a smaller but significant increase in RPA and RAD51 foci on chromatin of 2102EPcis-r cells after 16 h from drug washout.

As a second test for the efficiency of HR, we used the DR-GFP reporter system, which measures the error-free HR of a site-specific DSB formed by the rare-cutting endonucleases I-*Sce*I [37]. In this reporter, HR repair of the I*Sce*I-induced DSB leads to restoration of a GFP^+^ gene that uses iGFP as the template (Figure S4C). We observed that in GCT27cis-r cells, the proficiency in HR repair was increased by twofold relative to GCT27cis-s cells (Figure 2C). We also observed a significant increase in HR in 2102EPcis-r cells, although with a reduced magnitude (Figure 2D). As a third test for the efficiency of HR, we tested GCT27 and 2102EP paired cell lines’ sensitivity to the PARPi AZD2281, a drug that selectively kills cells that are deficient in HR [38]. We subjected cell lines to a chronic treatment with increasing doses of AZD2281 and evaluated their survival by colony assay. As shown in Figure 2E,F, PARPi sensitivity was reduced in cis-r cells compared to their cis-s counterparts, with a greater effect observed in GCT27cis-r cells. Next, we quantified the proficiency in ICL-induced HR by using the TR-OriP-GFP reporter. In this assay, the ICL is achieved by using a triplex-forming oligonucleotide conjugated to psoralen, and repair by HR of the DSBs generated from the processing of the ICL results in GFP^+^ cells [28,39] (Figure S4D). We measured a significant increase in the percentage of GFP^+^ cells in GCT27cis-r cells (Figure 2G), indicating that in resistant cells, HR contributed significantly to the repair of ICL-induced DSBs. Conversely, proficiency in ICL-induced HR was increased only mildly in 2102EPcis-r cells (Figure 2H). This suggests the involvement of additional mechanisms for the acquired resistance of 2102EPcis-r cells (see below and discussion).

### 3.4. Analysis of the Expression and Binding onto Chromatin of HR-Repair Factors

**Figure 3:**
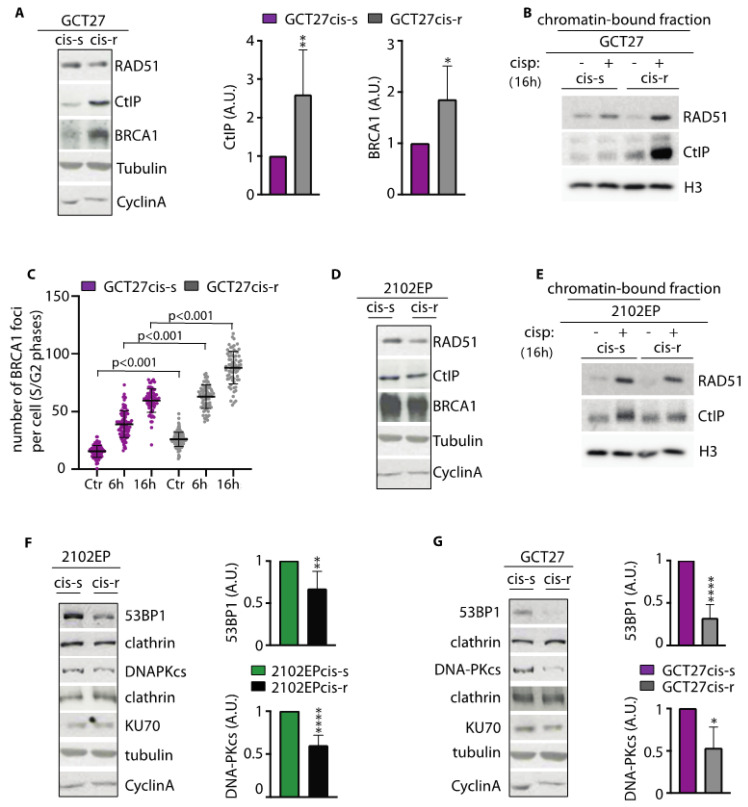
(**A**) Western blotting analyses of the indicated pro-homologous recombination (HR) factors in total cell extracts from GCT27 paired cell lines. Graph bars quantify protein level differences among GCT27 cell lines. Data are mean values ± s.d. of either three (breast cancer type 1 susceptibility protein, BRCA1) or six (C-terminal binding protein-1 interacting protein, CtIP) independent experiments. Original blots see Figure S3C. Statistical analyses were performed using a two-tailed *t*-test (* *p* < 0.05; ** *p* < 0.01). (**B**) Western blotting analyses of the indicated pro-HR factors in chromatin extracts from GCT27 paired cell lines. Cells were treated with a pulse of 3 μM cisplatin for 6 h and analyzed 16 h after culture in drug-free media. Histone H3 (H3) was used as a loading control. Original blots see Figure S3D. (**C**) Quantification of BRCA1 foci number in GCT27 paired cell lines before (Ctr) and after treatment with 3 μM cisplatin. Treated cells were collected both at the end of treatment (6 h) and 16 h after drug washout. Data are mean values ± s.d. of two independent experiments, each performed in duplicate. (**D**) Western blotting analyses of the indicated pro-HR factors in total cell extracts of 2102EP paired cell lines. Tubulin and cyclin A were assessed to detect differences in protein loading and cell cycle phase distribution, respectively. Original blots see Figure S3C. (**E**) Western blotting analyses of the indicated pro-HR factors in chromatin extracts from the indicated cell lines, before and after treatment with 3 μM cisplatin for 6 h. Cell extracts were prepared from cells collected 16 h after drug removal. H3 was used as a loading control. Original blots see Figure S3D. (**F**,**G**) Western blotting analyses of the indicated pro-non-homologous end joining (NHEJ) factors in total cell extracts of 2102EP and GCT27 cell lines. Clathrin was used as a loading control for 53BP1 and DNA-PKcs, while KU70 expression was normalized using tubulin. Graph bars quantify protein levels difference among cell lines. Original blots see Figure S3E. Data are mean values ± s.d. of six (53BP1) or three (DNA-PKcs) independent experiments. In all quantifications, the expression of the indicated proteins was normalized on the expression of either tubulin or clathrin. Cyclin A was assessed to detect possible differences in cell cycle phase distribution among cell lines. Statistical analyses were performed using a one-tailed *t*-test (* *p* < 0.05; ** *p* < 0.01; **** *p* < 0.0001). A.U. = arbitrary units.

To probe the molecular mechanism behind the HR response in GCT27 cells, we analyzed, using Western blotting, the basal expression of two key HR factors: RAD51 and CtIP. We observed that while RAD51 was evenly expressed across cell lines, the expression of CtIP was increased in GCT27cis-r cells (Figure 3A). The immunochemical analysis of a chromatin-enriched fraction showed that 16 h after cisplatin treatment, CtIP and RAD51 were bound onto chromatin at higher levels relative to GCT27cis-s cells (Figure 3B). Another protein with a key function in HR is BRCA1, which extends DNA resection, thus promoting the formation of an ssDNA tail that subsequently becomes the docking site of RPA and RAD51 [40]. Using Western blotting, we observed that BRCA1 basal expression was higher in GCT27cis-r cells (Figure 3A). To understand if such increased expression could have functional implications, we quantified the BRCA1 foci number upon cisplatin exposure of GCT27 paired cell lines. We observed that GCT27cis-r cells could assemble a significantly greater number of BRCA1 foci onto DNA, both in untreated and treated cells (Figure 3C). Given the function of CtIP and BRCA1 in DNA resection, we concluded that the increased proficiency in repair of I*Sce*I-induced and ICL-induced DSBs in GCT27cis-r cells likely relies on the improved activity of CtIP and BRCA1, with consequent increased binding of RPA and RAD51 to ssDNA.

The analyses of HR proteins’ basal expression and binding onto chromatin in 2102EP paired cell lines revealed no significant differences across cell lines (Figure 3D,E). This indicates that the observed increase in RPA/RAD51 loading and HR repair proficiency observed in these cell lines are not linked to overexpression of such HR proteins.

### 3.5. In cis-r Cell Lines 53BP1 and DNA-PKcs Expressions Are Dampened and NHEJ Is Attenuated

In mammalian cells, DSBs are repaired by two major pathways: NHEJ and HR. A key pro-NHEJ factor is 53BP1. Loss of 53BP1 alleviates HR repair defects and overcomes PARPi sensitivity of BRCA1-deficient cells by restoring DNA end resection and RAD51 loading [20,41]. In search of the molecular mechanisms behind the increased proficiency in HR of 2102EPcis-r cells, we investigated 53BP1 protein expression by Western blotting. We observed a significant reduction in 53BP1 protein levels in 2102EPcis-r cell lines, compared to their sensitive counterpart (Figure 3F). Moreover, by evaluating the expression of additional pro-NHEJ factors, we observed downregulation of DNA-PKcs in 2102EPcis-r cells, while the pro-NHEJ factor KU70 was evenly expressed across cell lines (Figure 3F). Interestingly, extending the analysis to GCT27 paired cell lines, we observed that 53BP1 and DNA-PKcs expression were also downregulated in the GCT27cis-r cell line (Figure 3G). This reduced expression could not be attributed to changes during cell cycle progression, as cell extracts showed an equivalent basal expression of cyclin A (Figure 3F,G).

**Figure 4:**
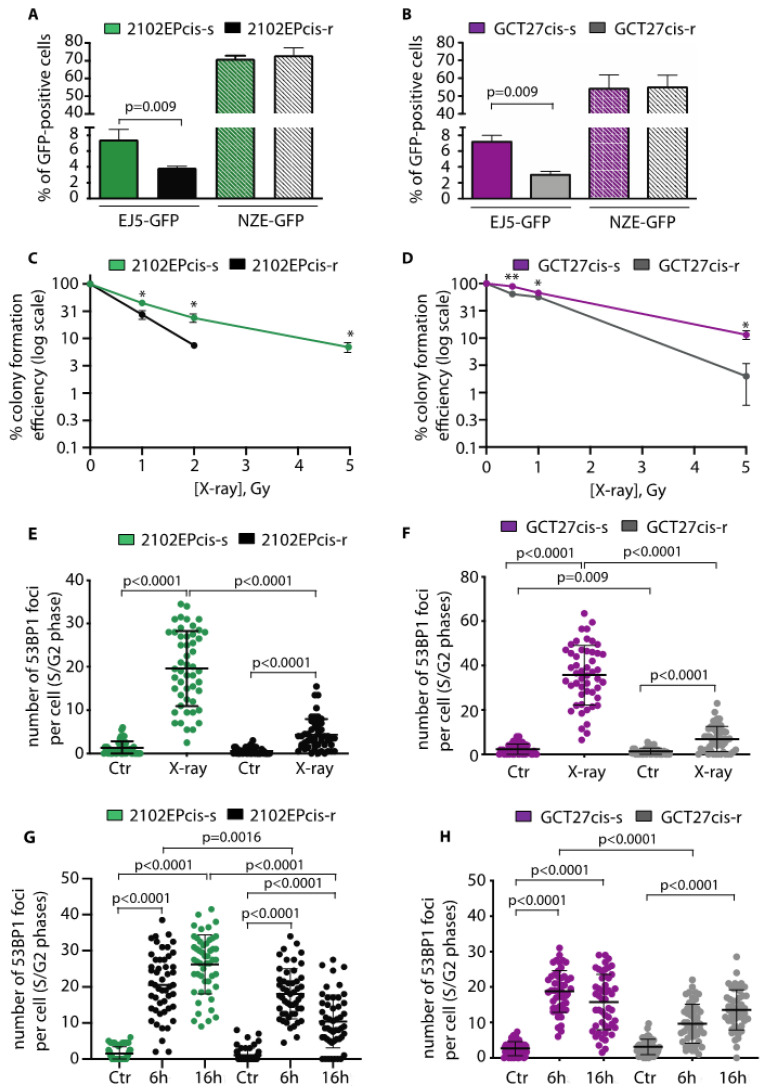
(**A**,**B**) NHEJ repair proficiency of GCT27 and 2102EP cell lines as measured by the EJ5-GFP reporter substrate. NZE-GFP indicates the transfection efficiency of each cell line. Data are mean values ± s.d. of three independent experiments. (**C**,**D**) Colony assay of the indicated cell lines treated with the indicated doses of X-rays. The surviving fraction was monitored by following colony formation for up to 10 to 14 days after treatment. Data are mean values ± s.d. of two independent experiments, each performed in triplicate. (**E**,**F**) Quantification of 53BP1 foci in S/G2 (cyclin A-positive) nuclei of the indicated cell lines after X-ray treatment. Data are mean values ± s.d. of three independent experiments. (**G**,**H**) Quantification of 53BP1 foci in S/G2 nuclei of the indicated cell lines after cisplatin treatment. Cells were treated with a 6 h pulse of 3 μM cisplatin and collected at the end of stimulation and 16 h after drug washout. Data are mean values ± s.d. of three independent experiments. Statistical analyses were performed using an unpaired two-tailed (**A**,**B**,**E**–**H**) or one-tailed (**C**,**D**) *t*-test (* *p* < 0.05; ** *p* < 0.01).

To explore whether the observed downregulation of 53BP1 and DNA-PKcs could affect DSB repair by NHEJ, we exploited the EJ5-GFP reporter substrate, which provides a measure of the proficiency in repair of I*Sce*I-induced DSBs by NHEJ (see [29] and Figure S4E). We found that NHEJ proficiency was reduced in both 2102EPcis-r and GCT27cis-r cells compared to their cis-s counterparts (Figure 4A,B). NHEJ-deficient cells are typically sensitive to DNA damage induced by ionizing radiation (IR) [14,19]. Thus, as a second test of NHEJ proficiency, we compared the X-ray sensitivity of paired cell lines using a colony forming assay. As shown in Figure 4C,D, both cis-r cell lines were relatively sensitive to IR, confirming a functionally relevant reduction in NHEJ. NHEJ represents the major DSB repair pathway in the repair of IR-induced DSBs in G2 [42]. Thus, to understand if the sensitivity of the cell lines to X-rays was linked to reduced 53BP1 function, we quantified 53BP1 foci in S/G2 phase cells after exposure to 5 Gy of IR. We found that after IR, in both cis-r cell lines, 53BP1 foci were greatly reduced relative to their cis-s counterparts (Figure 4E,F). We concluded that in cis-r cells, proficiency in DSB repair by NHEJ in G2 is attenuated, likely because of the reduction in 53BP1 (and DNA-PKcs) protein levels.

### 3.6. Modulation of 53BP1 Protein Expression Level Alters 2102EP and GCT27 Cells Sensitivity to Cisplatin

To investigate whether the reduced assembly of 53BP1 during S/G2 might be relevant to 2102EPcis-r and GCT27cis-r response to cisplatin, we quantified 53BP1 foci after drug treatment. To this end, cis-s and cis-r paired cell lines were exposed to cisplatin for 6 h and S/G2-phase cells were stained with anti-53BP1 antibody. In the 2102EPcis-r cell line, we observed a reduction in the average number of 53BP1 foci at both time points (Figure 4G), as well as 53BP1 association with chromatin (Figure S4F). Similarly, a reduction in foci and binding onto chromatin was observed in GCT27cis-r cells after 6 h of treatment (Figure 4H and Figure S4G). We also observed a reduction in 53BP1 foci in GCT27cis-r cells after drug release, although the difference was not statistically significant (Figure 4H).

**Figure 5:**
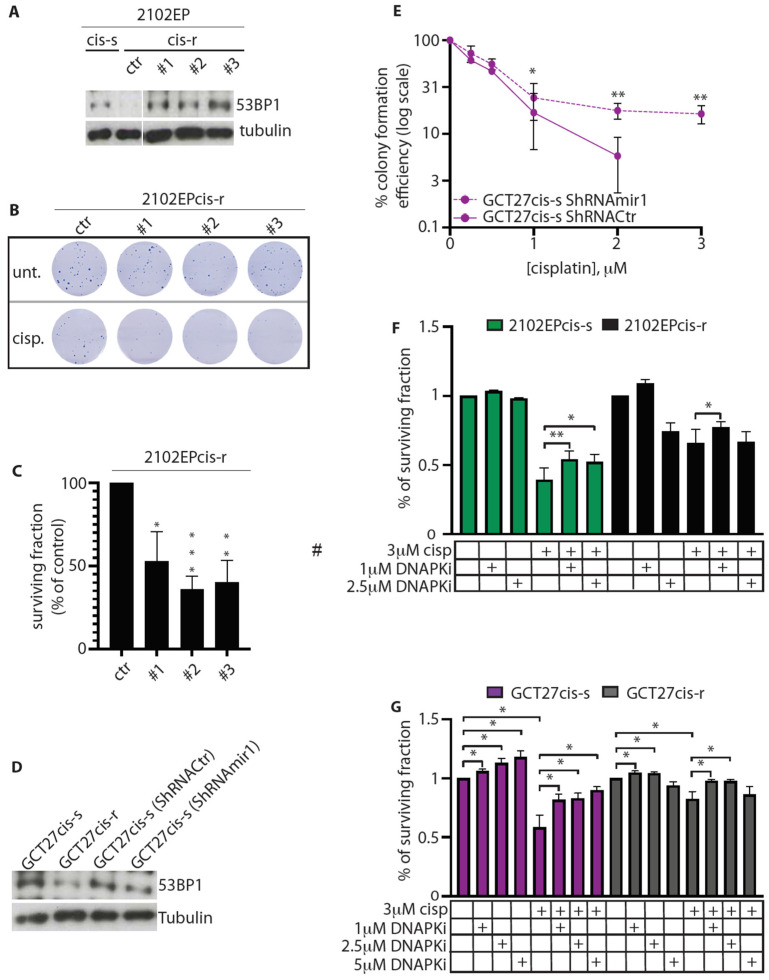
(**A**) Western blotting analyses of 53BP1 expression in 2102EP cells. Here, #1, #2, #3 are clones of 2102EPcis-r cells overexpressing 53BP1 at a level comparable to that of cis-s cells. Ctr indicates the level of 53BP1 protein in naïve 2102EPcis-r cells. Tubulin was used as loading control. Original blots see Figure S3F. (**B**) Colony assay of control and 53BP1-overexpressing clones of 2102EPcis-r after treatment with a pulse of 3 μM cisplatin for 6 h. Ctr = cells transfected with a control vector; Unt. = untreated; cisp. = cisplatin-treated. (**C**) Quantification of surviving colonies of the indicated 2102EPcis-r cells, treated as described in (**B**). (**D**) Western blotting analyses of 53BP1 expression in GCT27 cells. GCT27cis-s and GCT27cis-r are non-infected paired clones. GCT27cis-s ShRNACtr are cells infected with a non-specific shRNA, while GCT27cis-s ShRNAmir1 are cells infected with an shRNA specific to 53BP1. Tubulin was used as a loading control. Original blots see Figure S3F. (**E**) Colony assay of GCT27 cell lines infected with either ShRNACtr or ShRNAmir1. Data are mean values ± s.d. of three independent experiments. (**F**,**G**) Surviving fraction of the indicated cell lines after treatment with either cisplatin or cisplatin combined with DNA-PKi. Survival was evaluated by crystal violet assay. Data are mean values ± s.d. of either three (GCT27) or six (2102EP) independent experiments. Statistical analyses were performed using the unpaired two-tailed (**C**,**F**,**G**) or one-tailed (**E**) *t*-test (* *p* < 0.05; ** *p* < 0.01; *** *p* < 0.0001).

These results suggest a correlation between reduced 53BP1 expression/assembly in S/G2 and acquired resistance to cisplatin. This assumption predicts that modulation of 53BP1 expression level should alter the response of cell lines to cisplatin. To test this hypothesis, we increased 53BP1 expression in 2102EPcis-r cells using a retroviral vector. Overexpression was lethal for most cells. However, using long-term cell culture, we could isolate three clones whose 53BP1 protein level was comparable to that of 2102EPcis-s cells (Figure 5A) and tested their sensitivity to cisplatin. As shown in Figure 5B and quantified in Figure 5C, we found that upregulation of 53BP1 in 2102EPcis-r cells increased their sensitivity to cisplatin by approximately two- to threefold compared to 2102EPcis-r transfected with a control vector (Ctr).

Unfortunately, we were unable to generate clones of GCT27cis-r cells overexpressing 53BP1, likely due to a greater toxic effect. Therefore, we investigated whether dampening of the 53BP1 protein level in GCT27cis-s cells could induce cisplatin resistance. We infected GCT27cis-s cells with shRNAmir1, which downregulates the 53BP1 protein to a level similar that detected in GCT27cis-r cells. As a control, we used an shRNAmir (ShRNACtr) that had no specific effect on 53BP1 (Figure 5D). After infection, cell lines were subjected to a pulse of cisplatin for 6 h, and their resistance was assessed by colony assay. As shown in Figure 5E, silencing of 53BP1 in GCT27cis-s cells significantly increased their survival following exposure cisplatin at all tested doses of the drug. GCT27cis-s cells were also silenced using a second shRNAmir (shRNAmir2) that decreases 53BP1 expression to an almost undetectable level (Figure S4H). Under this experimental condition, we observed that even though silenced GCT27cis-s cells demonstrated a greater resistance to 1 and 2 μM cisplatin doses, they did not survive upon exposure to cisplatin concentrations above 2 μM (Figure S4I). Taken together, these findings demonstrate that acquired cisplatin resistance is dependent on a specific and narrow range of reduction in 53BP1 expression levels.

### 3.7. Inhibition of DNA-PKcs Activity Reduces the Cytotoxic Effect of Cisplatin

If the reduction in DNA-PKcs expression in 2102EPcis-r and GCT27cis-r cells functions to increase cisplatin resistance by reducing DNA-PKcs activity, inhibition of DNA-PKcs by the DNA-PKcs inhibitor (DNA-PKi) NU7441 [43] should reduce cisplatin cytotoxicity. To verify this hypothesis, we pre-treated cells with a pulse of 3 µM cisplatin for 6 h and, after a washout, cultured them in a continuous administration with increasing concentrations of NU7741 for 72 h. Crystal violet assay was used to evaluate cell survival. Consistent with our hypothesis, we observed that in 2102EPcis-s cells, DNA-PKi treatment reduced cisplatin cytotoxicity at both 1 and 2.5 μM concentrations (Figure 5F). The addition of DNA-PKi also increased cell survival in GCT27cis-s cells at concentrations up to 5 µM (Figure 5G). Interestingly, treatment of 2102EPcis-r and GCT27cis-r cells (which have a reduced 53BP1 protein level) with 2.5 and 5 µM DNA-PKi doses, respectively, did not reduce cisplatin cytotoxicity (Figure 5F,G). This suggests that for the acquisition of cisplatin resistance, DNA-PKcs activity reduction must occur within a narrow range, which changes with the level of basal expression of the protein.

To further test the consequence of combining cisplatin and DNA-PKi, we compared the effect of the combination with that of each single drug using the combination index (CI), where CI < 1, CI = 1 and CI > 1 indicated synergistic, additive and antagonistic effects, respectively [44]. We found that DNA-PKi exerted an antagonistic effect on cisplatin in both cis-and cis-r cells (Table S1).

### 3.8. Expression of TP53BP1 and PRKDC in Non-Seminomas Patients

To analyze whether low expression of 53BP1 and DNA-PKcs correlates with tumor progression/recurrence in patients with non-seminoma GCTs, we performed in silico analyses using a public RNAseq database (see Material and Methods). Among non-seminomas, we restricted our analyses to ECs, teratomas (known to be relatively insensitive to chemotherapy [45,46]) and mixed GCTs containing EC components. We observed that *TP53BP1* expression was reduced in patients with worse progression-free survival (Figure S5A), although the difference was not statistically significant. In humans, the DNA-PKcs enzyme is encoded by the gene designated as *PRKDC*. The analysis of *PRKDC* expression in the same subset of tumors revealed that high DNA-PKcs expression correlated with tumor progression/recurrence (Figure S5B). From these observations, we concluded that in vivo, tumor progression and recurrence after primary treatment might benefit from a reduction in 53BP1 protein level, while, concomitantly, the expression of DNA-PKcs remains sustained.

### 3.9. In Cis-r Cells PARP Inhibition Has an Additive/Synergistic Interaction with Cisplatin

**Figure 6:**
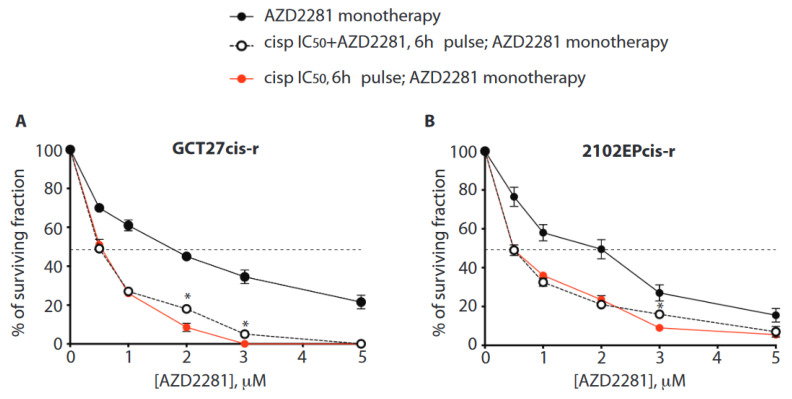
(**A**,**B**) Colony forming assay of the indicated cell lines treated with cisplatin and poly (ADP-ribose) polymerase inhibitor (PARPi) (AZD2281) combined therapy. AZD2281 monotherapy: Cells were treated with the indicated doses of AZD2281 as a single agent. Cisp IC_50_+ AZD2281, 6 h pulse; AZD2281 monotherapy: Cells were treated with the IC_50_ dose of cisplatin in combination with the indicated doses of AZD2281 for 6 h. After a washout, cells were incubated with the indicated concentrations of PARPi. Cisp IC_50_, 6 h pulse; AZD2281 monotherapy: Cells were treated with the IC_50_ dose of cisplatin for 6 h. After a washout, they were incubated with the indicated increasing concentrations of PARPi as a single agent. Data are mean values ± s.d. of two independent experiments, each performed in triplicate. Combined treatments were both statistically different compared to PARPi monotherapy, at all PARPi amounts tested (*p* < 0.05); unpaired one-tailed *t*-test. Asterisks (*) indicate where a statistically significant difference was found comparing the two cisplatin/AZD2281 co-treatment schedules (unpaired one-tailed *t*-test, * *p* < 0.05). The dotted line intercepts the half-maximal inhibitory concentration (IC_50_).

By studying a set of non-isogenic EC testicular tumor cell lines, we previously demonstrated that response to cisplatin monotherapy could be enhanced by combined treatment with the PARPi AZD2281 [11]. Here, we asked whether combined therapy could impact the cell survival of tumor cells with acquired resistance. In the clinic, cisplatin toxicity is a concern, especially when it is administered in high cumulative doses, as in patients who receive cisplatin as part of both first-line and salvage chemotherapy [1]. Conversely, AZD2281 has limited toxicity in patients [47] and in recombination-proficient cells, such as the non-tumorigenic breast epithelial cell line MCF10A, treated with clinically achievable doses (Figure S6A) [48]. Thus, we aimed to identify the dose of AZD2281 able to promote chemotherapy response in GCT cis-r cells treated with a reduced concentration of cisplatin. To this end, increasing doses of AZD2218 were given in combination with an IC_50_ dose of cisplatin. To evaluate which administration schedule is most efficient, we compared two alternative conditions. In the first setting, cells were pre-treated with a pulse of IC_50_ of cisplatin for 6 h and successively exposed to AZD2281 monotherapy. In the second setting, cells were given a pulse of cisplatin IC_50_ in combination with increasing doses of AZD2281 and successively exposed to AZD2281 monotherapy. We evaluated cell survival by colony assay. This experiment revealed that in both experimental settings, AZD2281 enhances the cytotoxicity of cisplatin in both cis-r cell lines (Figure 6A,B).

Among cell lines, we observed a greater effect in the GCT27cis-r cells, as indicated by the IC_90_ value for AZD2281 (Table 3).

Importantly, the analysis of drugs’ interaction revealed that when AZD2281 was given at a dose of 1 µM (a sub-lethal dose in recombination-proficient cells [48], Figure S6A), the drugs interacted additively, while the interaction was synergistic at concentrations ≥ 2 µM, regardless of the administration schedule (Table 4). Finally, we tested whether pre-treatment with AZD2281 could enhance the effect of cisplatin/AZD2281 combined therapy. We did not observe any further therapeutic advantage (Figure S6B,C).

## 4. Discussion

To date, the two major salvage approaches used to cure GCT patients that fail to achieve a durable response to first-line treatment include conventional-dose chemotherapy and high-dose chemotherapy with autologous stem cell transplant, but unfortunately, these strategies are only successful approximately 50% of the time [1]. Understanding the molecular mechanisms underlying GCT resistance to cisplatin is therefore essential to identify new targeted therapies.

In the present work, we show that both GCT27cis-r and 2102EPcis-r cell lines have increased proficiency in repairing ICL-induced DSBs compared to their cis-s counterparts. This occurs through the employment of conservative DNA repair mechanisms, as indicated by the improved genome stability in cis-r cells subjected to cisplatin treatment. This observation is consistent with our previous finding that sensitivity of testicular EC cell lines to cisplatin correlates with reduced usage of HR [11] and with the previously reported hyper-methylation of promoters of the pro-HR factors BRCA1 and RAD51c in non-seminomas [49,50]. Nevertheless, DSBs that form after ICL damage are not immediately available for HR, as the homologous template is first processed by multiple repair mechanisms. These include FANCD2-dependent endonuclease cleavage, which is a potential target of cisplatin resistance [12]. Here, we showed that in the cis-r cells under study, FANCD2 expression is upregulated, and that in the GCT27cis-r cell line, the FANCD2 pathway is hyper-activated after cisplatin treatment, as indicated by the cells’ ability to overcome psoralen-induced damage in the ICL repair assay. Moreover, we also observed increased efficiency in DSBs’ repair by HR, and this correlates with increased expression and binding onto chromatin of RAD51 and CtIP and loading of RPA and BRCA1 after cisplatin-induced DNA damage. These data suggest that both FA and HR pathways are involved in the acquisition of cisplatin resistance in this cell line. Importantly, we also observed a reduction in 53BP1 protein expression as well as in its ability to bind cisplatin-damaged chromatin. Previous works have shown that downregulation of 53BP1 expression rescues RAD51 loading in cells with suboptimal BRCA1 function [51] and increases cisplatin resistance in cells with wild-type BRCA1 [20,21]. Thus, we propose that dampening of the 53BP1 protein level contributes to the improved proficiency in HR repair of GCT27cis-r cells compared to their cis-s counterpart.

The levels of 53BP1 protein and its ability to bind chromatin are also reduced in 2102EPcis-r cells, suggesting that a similar mechanism of acquired resistance also occurs in this cell line. In agreement with this hypothesis, resection of DSBs (as measured by loading of RPA and RAD51 foci) and HR repair proficiency are significantly increased in 2102EPcis-r cells compared to their cis-s counterpart. Nonetheless, the ability of 2102EPcis-r cells to overcome psoralen-induced DSBs by HR is only mildly increased. Analysis of FANCD2 expression and activation in 2102EPcis-r cells reveals that although the FANCD2 basal level is increased compared to 2102EPcis-s cells, this does not result in an enhanced ability to bind chromatin and assemble into foci, indicating a lack of functional implementation of the FA pathway. Since Fanconi Anemia Complementation Group (FANC) proteins act on ICL intermediates in advance of HR, we can conclude that the FA pathway is defective in both 2102EPcis-r and 2102EPcis-s cells, thus limiting the usage of HR in the ICL repair assay.

Genetic studies have shown that downregulation of NHEJ proteins KU70/KU80 or downregulation or inactivation of DNA-PKcs rescues the cisplatin sensitivity of FA defective cells. This is likely due to the inappropriate joining of ICL-induced DSBs with other DSBs present in the cell when NHEJ is active [19,20]. Remarkably, we found that in both GCT27 and 2102Ep cis-r cell lines, NHEJ proficiency is impaired, and this correlates with a reduced expression of DNA-PKcs. The contribution of DNA-PKcs activation to cisplatin resistance is confirmed by inhibition studies showing that the addition of a DNA-PKi in culture reduces cisplatin cytotoxicity. Interestingly, the dose of DNA-PKi required to reduce the cytotoxicity of cisplatin appears to vary accordingly to the basal expression level of DNA-PKcs, with cis-r cells requiring lower dosages than cis-s cell lines. Moreover, the exposure of cis-r cells to a higher dose of the inhibitor does not result in a further reduction in cisplatin cytotoxicity. These findings suggest that a fine tuning of DNA-PKcs expression/function is crucial for the acquisition of cisplatin resistance. This conclusion agrees with the finding that recovery of the ICL sensitivity of FA cells varies with the dose of DNA-PKi [19,22].

In this study, we also demonstrated the contribution of 53BP1 in the acquisition of resistance to cisplatin through the modulation of its expression. Silencing of 53BP1 in GCT27cis-s cells increases their cisplatin resistance, whereas increased 53BP1 expression in 2102EPcis-r cells sensitizes them to cisplatin. Interestingly, excessive silencing of 53BP1 in GCT27cis-s cells makes them sensitive to doses of cisplatin >2 µM. This highlights that a narrow and specific range of expression of this DNA repair factor is integral for the acquisition of cisplatin resistance in this cell line. It is possible that if the capacity of 53BP1 to bind damaged chromatin is reduced beyond a certain threshold, DSB resection could shift to hyper-resection, which, in turn, might cause repair of DSBs through a mutagenic pathway [52]. Rescue of cisplatin sensitivity by reduced expression/function of 53BP1 was surprising since previous studies have shown that its downregulation in BRCA1-deficient cells increases HR repair proficiency without reducing sensitivity to ICL-inducing agents [20]. However, since in our cis-r cells, BRCA1 was expected to be functional, the present results suggest that the effect of 53BP1 downregulation on cisplatin-induced resistance relies on the specific context of DNA repair factor expression in the cell. Indeed, a reduced function of 53BP1 has been shown to correlate with resistance to mitomycin C in FA deficient cells and tumors with functional BRCA1 [22,53].

The hypothesis that reduction in 53BP1 expression is involved in the acquisition of cisplatin resistance is supported by the in silico finding that patients with the worst disease-free survival tend to show low expression of *TP53BP1*. This suggests a role for 53BP1 downregulation in tumor recurrence in a clinical setting. However, *PRKDC* expression is not concomitantly reduced in this cohort of patients. Possibly, in vivo, the expression of pro-NHEJ factors is modulated to avoid an excessive reduction in NHEJ. This agrees with our observation that excessive downregulation of NHEJ is not beneficial in cells under the selective pressure of cisplatin.

Based on our findings, we therefore propose that naïve EC cells are poorly HR (and FA)-proficient [11], and that under cisplatin selective pressure, resistance is acquired by a fine-tuned modulation of the DNA repair pathways. The reduced expression of 53BP1 (as well as the increased expression and activity of CtIP and BRCA1 in GCT27cis-r cells) is likely to promote DSB resection, with a consequent increase in HR repair proficiency and PARPi resistance. In addition, the concomitant downregulation of NHEJ by reduction in 53BP1 and DNA-PKcs functions restrains NHEJ-mediated mutagenic repair of the ICLs [20,21], thus promoting maintenance of genome stability and survival after treatment.

A relevant observation of our studies is that due to the increased proficiency in HR, cis-r cells are relatively resistant to PARPi. This is in agreement with a recent finding from a phase II trial in which AZD2281 demonstrated marginal activity as a single agent in cisplatin-refractory patients [54]. Importantly, here, we show that cells relatively resistant to either cisplatin or PARPi monotherapies are highly sensitive to combination therapy, even if cisplatin is administered at the IC_50_ dose, i.e., two- to threefold lower than the median plasma concentration measured in testicular tumor patients treated with cisplatin [55]. The effect of combined treatment in EC cell lines correlates with an increased load of DNA damage [11], likely caused by genomic rearrangements induced by the combination [11,48]. At the basis of the combinatorial effect, there might be the downregulation, caused by AZD2281, of HR protein expression, with consequent sensitization to cisplatin-induced damage [56,57].

Remarkably, by comparing three administration schedules, we showed that sequential cisplatin-pulse>AZD2281 monotherapy treatment strongly reduces the survival of cis-r cells, to an extent that is similar to when drugs are administered concomitantly. This suggests that concurrent administration is unnecessary. We can speculate that pre-treatment with AZD2281 does not give any further therapeutic advantage. Such findings provide a rationale for the conduct of future clinical trials combining a PARP inhibitor with cisplatin in cisplatin-refractory patients. In addition, our findings raise the possibility that drugs stabilizing or enhancing 53BP1 or DNA-PKcs levels or function could also have activity in cisplatin-refractory GCT.

We believe that these results provide significant insights into the mechanisms of acquired resistance of testicular tumors to cisplatin, with important potential clinical implications.

## 5. Conclusions

Since the introduction of cisplatin-based chemotherapy in the mid-1970s, GCTs have been cured with unusually high success. However, a relevant number of (typically young) patients still require salvage therapy, which is only successful in 50% of cases. Here, we characterized a mechanism of cisplatin-acquired resistance through which GCT cancer cells under cisplatin treatment selection adapt their recombinative abilities. Cells with increased drug resistance improve their ability to repair cisplatin-induced damage by HR and, in some cases, upregulate the FA pathway. Concomitantly, cells dampen the efficiency of the NHEJ pathway by reducing the expression of 53BP1 and DNA-PKcs, thus constraining the toxic effect of NHEJ-mediated repair. Adaptation of DNA repair capabilities in cis-r cells reduces genome instability upon cisplatin exposure, thus improving their survival.

Further studies are needed to determine whether the mechanisms that we have described using the cellular system occur in vivo. In such a case, it will be possible to implement patient stratification to direct them toward more effective therapies.

Investigation on the response of cis-r cells to treatment with a PARP inhibitor revealed that, beside resistance of these cells in monotherapy, the PARPi AZD2281 exerted an additive/synergistic effect when combined with cisplatin, suggesting the possibility of introducing PARPi(s) in salvage therapy of patients. In this regard, prospective clinical trials will have to be designed to ensure that the combined treatment has effects less deleterious than those induced by the use of high doses of cisplatin employed nowadays in salvage therapy.

## Figures and Tables

**Table 1:** List of the antibodies, commercial sources and dilutions used in this study.

Antibodies	Source	Code	Working Solution
RAD51 (H-92)	Santa Cruz	Sc-8349	1:500
BRCA1 (C-20)	Santa Cruz	Sc-642	1:250
CtIP	Bethyl Laboratories	A300-488A-T	1:1000
KU70	Novus Biologicals	NB 100-1915	1:2000
DNAPKcs	Thermofisher	MA5-13244	1:200
TP53BP1	OriGene	TA309918	1:1000
FANCD2	Novus Biologicals	NB 100-182	1:1000
TUBULIN	Sigma-Aldrich	T4026	1:15,000
CLATHRIN	BD Bioscience	610500	1:1000
CYCLIN A (H-432)	Santa Cruz	Sc-751	1:1000

**Table 2:** Half-maximal inhibitor concentrations (IC_50_) and 90% of maximal inhibition (IC_90_) for cisplatin in the indicated cell lines. Data are mean values ± s.d. of at least three independent experiments. RF = resistance factor.

**6 h cisp 3 µM**	**GCT27** **cis-s IC_50_**	**GCT27** **cis-r IC_50_**	**2101EP** **cis-s IC_50_**	**2101EP** **cis-r IC_50_**
	0.31 ± 0.03	1.27 ± 0.11	0.29 ± 0.05	1.05 ± 0.22
RF		4		3.6
	**IC_90_**	**IC_90_**	**IC_90_**	**IC_90_**
	0.84 ± 0.02	4.91 ± 0.16	0.86 ± 0.08	5.07 ± 1.12
RF		5.8		5.9

**Table 3:** IC_90_ of AZD2281 in monotherapy (mono) (top row), in combined sequential (cisplatin>AZD2281) monotherapies (middle row) and combined therapy with concomitant administration of cisplatin and AZD2281, followed by AZD2281 monotherapy (bottom row). In all cases, cisplatin (cisp) concentration was equal to the IC_50_ value.

IC_90_ Value for AZD2281		
	GCT27 cis-r IC_90_ (µM)	2102EP cis-r IC_90_ (µM)
AZD2281 mono	15.7 ± 1.7	9.4 ± 1.2
6 h cisp IC_50_ + AZD2281 mono	1.7 ± 0.007	4 ± 0.6
6 h cisp IC_50_/AZD2281 + AZD2281 mono	2.4 ± 0.07	4.6 ±0.3

**Table 4:** Combination index (CI) of cisplatin/AZD2281 combined therapies. Top row: combined sequential (cisplatin>AZD2281) monotherapies (mono). Bottom row: concomitant administration of cisplatin and AZD2281 for 6 h, followed by AZD2281 monotherapy. Cisplatin (cisp) concentration was equal to the IC_50_ value; AZD2281 concentrations are indicated.

		GCT27 cis-r	2102EP cis-r
	**[AZD2281]**	**CI**	**CI**
**6 h cisp IC_50_ +**	1 µM	0.67	1.1
**AZD2281**	2 µM	0.28	0.93
**mono**	3 µM	0.04	0.46
	5 µM	0.05	0.35
	**[AZD2281]**	**CI**	**CI**
**6 h cisp**	1 µM	0.7	1
**IC_50_/AZD2281**	2 µM	0.58	0.85
**+ AZD2281**	3 µM	0.19	0.79
**mono**	5 µM	0.05	0.48

## Data Availability

The data presented in this study are available from the corresponding author on a reasonable request.

## Supplementary Materials

The following are available at https://www.mdpi.com/2072-6694/13/4/787/s1, Figure S1: colony formation efficiency upon treatment with DNA-damage agents, Figure S2: DNA damage repair dynamic, cell death and FANCD2 activation upon treatment with cisplatin, Figure S3: Original blots of Figure 1, Figure 2 and Figure 5, Figure S4: HR factors assembly, DR-GFP, Tr-GFP, EJ5 reporter substrates description, 53BP1 expression and survival of GCT27 cell lines upon silencing with mir2 ShRNAi, Figure S5: Expression of *TP53BP1* and *PRKDC* in patients with a different degree of progression-free survival, Figure S6: Dose-response survival curves of MCF10A and EC cell lines upon treatment with AZD2281 or AZD2281/cisplatin combined therapy. Table S1: Combination index of cells treated with cisplatin and DNAPKi, Figure S7: Original blots of Figures S2 and S4.
