# Supplementary material for: Testicular Germ Cell Tumors Acquire Cisplatin Resistance by Rebalancing the Usage of DNA Repair Pathways
Source: Cancers (Basel). 2021 Feb 13;13(4):787. doi: 10.3390/cancers13040787 (PMC7917736; doi:10.3390/cancers13040787)
Supplement: Supplementary file 1 [file cancers-13-00787-s001.pdf]

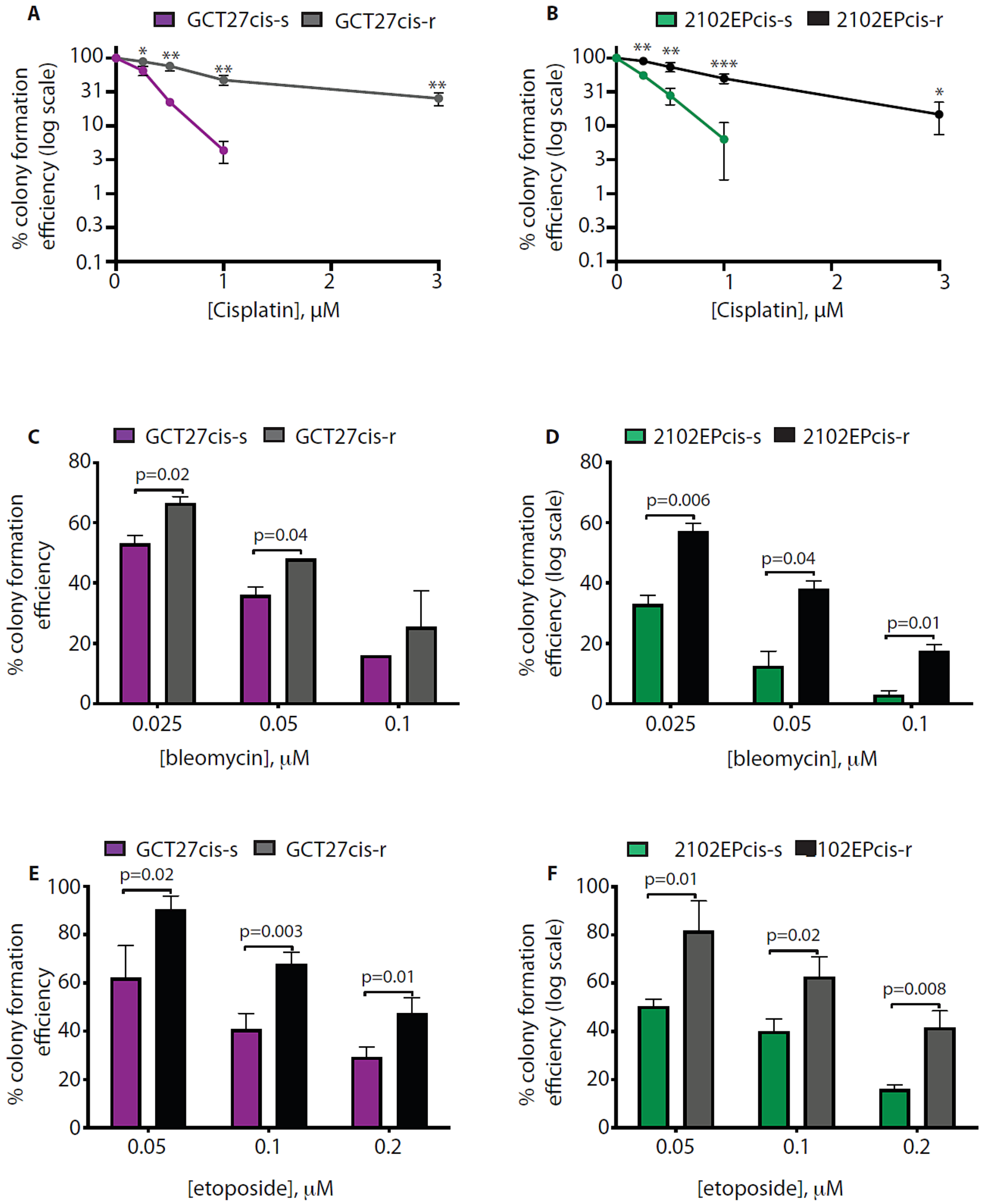

Figure S1. Colony formation efficiency upon treatment with DNA-damage agents. (**A**–**F**) Colony assay of the indicated EC cell lines, following treatment with DNA damaging agents. Cells were treated with increasing concentrations of the indicated drugs for 6 h, and after a wash out were cultured for additional 7–10 days and stained for quantification o colonies number. Data are mean value ± s.d. of three independent experiments in triplicates. Statistical analyses were performed using a two-tailed t-test ($p < 0.05$).

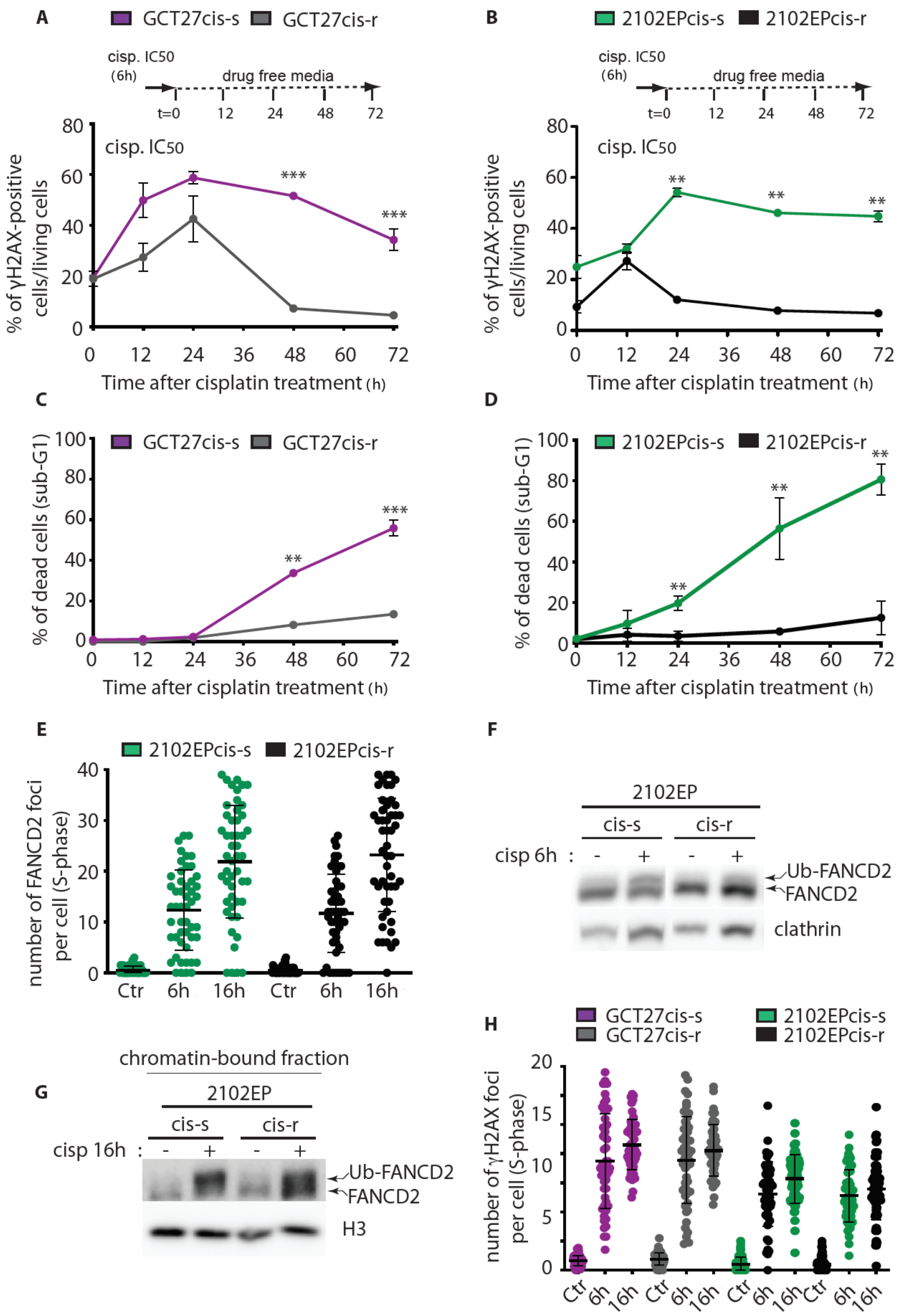

Figure S2. DNA damage repair dynamic, cell death and FANCD2 activation upon treatment with cisplatin. (**A**–**B**) Flow cytometry of H2AX Ser139 (γH2aX)-positive cells upon administration of the IC_{50} dose of cisplatin. EC cell lines were treated with the drug for 6hr and collected for the analyses at the indicated time points after cisplatin washout. Data are mean value ± s.d. of two independent experiments. (**C**–**D**) Quantification of sub-G1 fraction in the indicated cell lines. The cis-s EC cell lines are eliminated by apoptosis by 24 h or 48 h after treatment. The percentage of dead cells/total, following cisplatin treatment are shown. (**E**) Quantification of FANCD2 foci in 2102EP cells after 6hr pulse with 3 µM cisplatin. Cells were analyzed at the end of treatment (6 h) and after 16hr of culture in drug-free media. The authors observed no statistically significant differences between cell lines. Data are mean value ± s.d. of three independent experiments. Statistical analyses were performed using a two-tailed t-test. (**F**) Western blotting analyses of FANCD2 expression in 2102EP paired cell lines before and after treatment with 3µM cisplatin. Arrows point to the active monoubiquitinated (Ub-FANCD2) and non-monoubiquitinated inactive (FANCD2) forms of the protein. Original blots in Figure S7. (**G**) Western blotting analyses of FANCD2 in chromatin extracts of 2102EP cell lines before and after treatment with cisplatin. Cell were collected 16hs after drug washout. Original blots in Figure S7. (**H**) Quantification of γH2AX foci in BrdU-positive (S-phase) GCT27 and 2102EP cells, in absence and upon cisplatin treatment. Quantification was performed both at the end of treatment (6hr pulse with 3 µM cisplatin) and 16hr after drug washout. Data are mean value ± s.d. of three independent experiments.

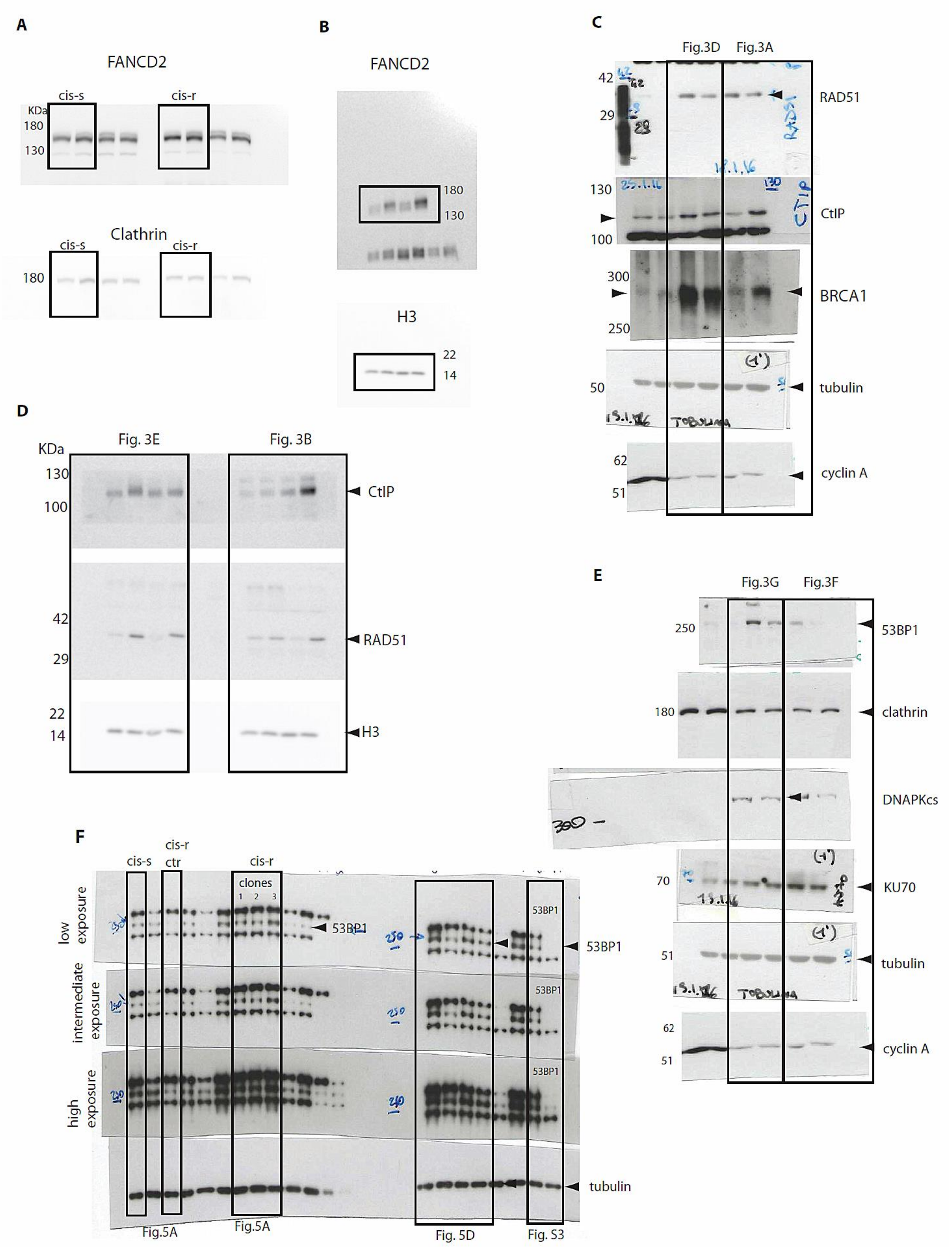

Figure S3. Original blots of Figures 1,2,5,S4.

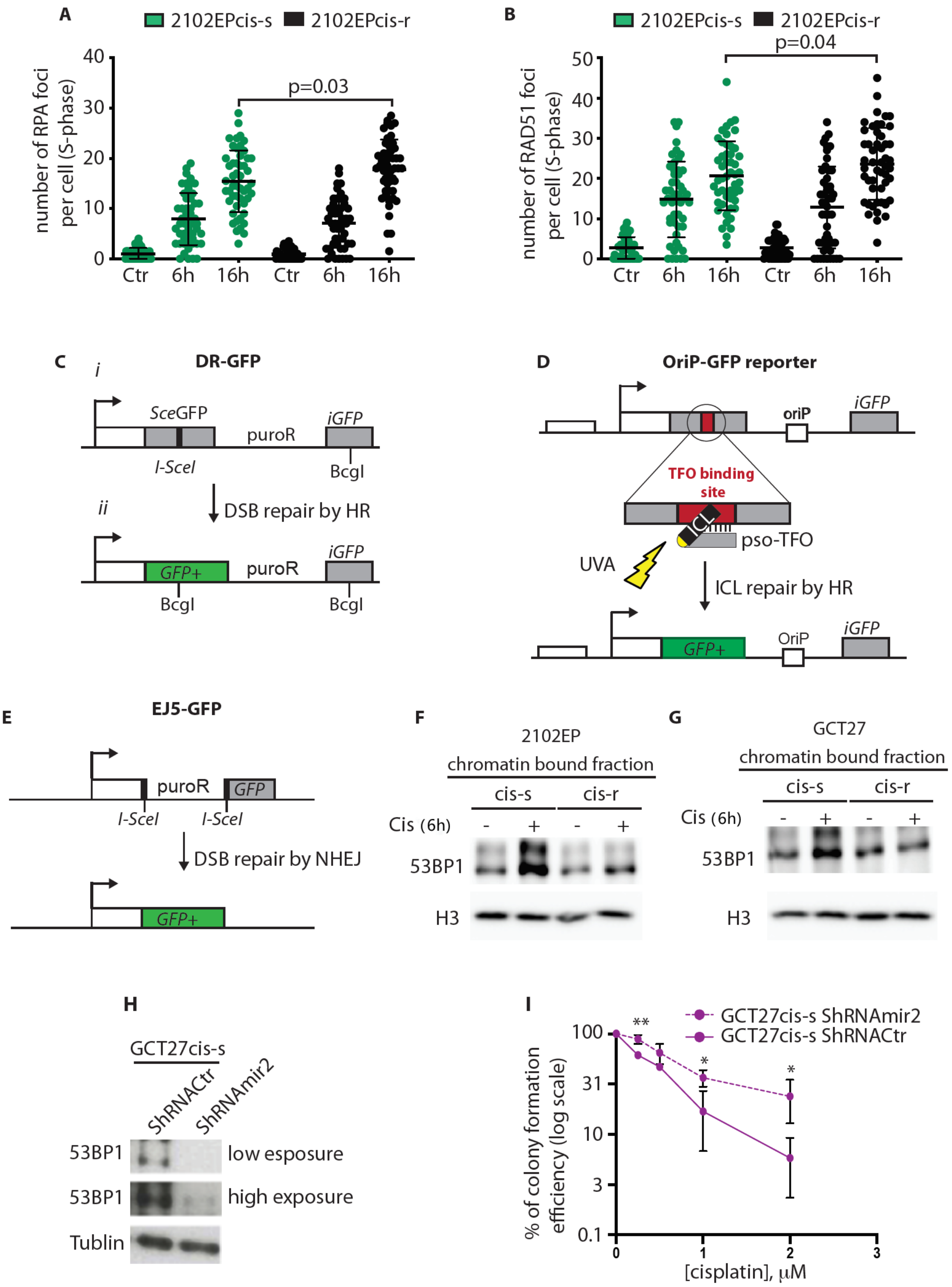

Figure S4. HR factors assembly, DR-GFP, Tr-GFP, EJ5 reporter substrates description and 53BP1 expression and survival in GCT27 cell lines silenced with mir2 ShRNAi. (**A**–**B**) Quantification of RPA and RAD51 foci in BrdU-positive 2102EP cells after 6 h pulse with 3 µM cisplatin. Cells were analyzed at the end of drug treatment (6 h) and 16 h after washout. Data are mean value ± s.d. of two independent experiments. (**C**) Diagram of the DR-GFP substrate used to measure HR. The DR-GFP gene is a modified green fluore*Sce*nt protein (*GFP*) gene in which GFP is modified to *SceGFP* so as to contain an I-*Sce*I site (incorporated at the BcgI site) and in frame termination codons. Downstream of the *SceGFP* gene, is an internal GFP fragment (*iGFP*). Electroporation of an I-*Sce*I expression vector will generate a DSB that can be repaired by several mechanisms, including HR, non homologous end-joining (NHEJ), or Single-strand annealing (SSA). Repair of DR-GFP substrate by HR restores GFP function if the *iGFP* sequence is used as the repair template. If this occurs, the cells become GFP-positive (GFP^{+}) and acquire green fluorescence which is quantified using FACS. In absence of expression of I-*Sce*I a DSB is not formed, and cells are GFP negative. (**D**) Diagram of the TR-OriP-GFP reporter used to measure ICL-induced HR. The TR-OriP-GFP reporter is a modified version of the DR-GFP substrate where the I-*Sce*I binding site has been replaced by a triplex-forming oligonucleotide (TFO) binding site (red rectangle). The latter binds a short single-strand

oligonucleotide conjugated to psoralen (pso-TFO). The reporter plasmid also contains an EBV origin of replication (OriP) placed between the GFP repeats (white boxes) to allow the plasmid to replicate in presence of EBNA1. After triplex formation with a pso-TFO followed by UVA irradiation, an ICL is formed between the substrate and pso-TFO oligonucleotide. When the reporter replicate (in presence of EBNA1) ICL-induced HR restores an intact *GFP* gene, giving rise to GFP^+ cells. When the triplex forming oligonucleotide lacks psoralen (TFO), the ICL is not formed. As consequence the ICL-induced HR is not induced, and the percentage of GFP^+ cells is greatly reduced (background level). (**E**) Diagram of the EJ5-GFP reporter substrate used to measure NHEJ. In this substrate the GFP gene is interrupted by a puromicine cassette flanked by two I*Sce*I cut sites that are in the same orientation. Following DSB formation by I*Sce*I, if the damage is repaired by NHEJ the cut site is restored and cells become GFP+. (**F**–**G**) Western blotting analyses of 53BP1 expression in chromatin cell extracts from the indicated cell lines, before and after treatment with cisplatin 3μM for 6hr. Histone H3 was used as loading control. Original blots in Figure S7. (**H**) Western blotting analysis of 53BP1 protein expression in the GCT27cis-s cell line silenced with the ShRNAmir2. The latter causes a strong down-regulation of protein expression, as shown by a weak signal only visible at high exposure. ShRNACtr is a non-specific siRNA. Original blot in Figure S3. (**I**) Colony assay of GCT27cis-s cells infected with ShRNACtr and ShRNAmir2. Strong silencing of 53BP1 increases cisplatin resistance of GCT27cis-s cells only up to 2 μM concentration. Data are mean value ± s.d. of two independent experiments in triplicates. Statistical analyses were performed using a one-tailed t-test. (**$p < 0.01$; ***$p < 0.001$).

A

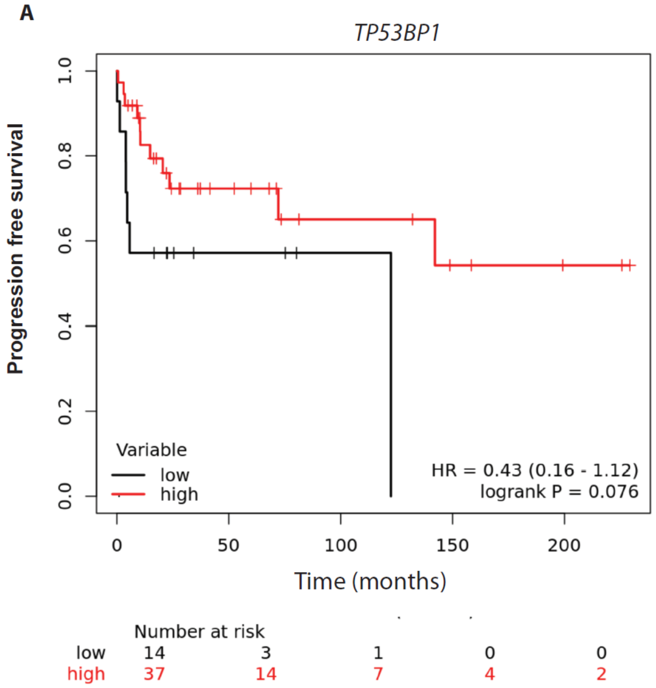

B

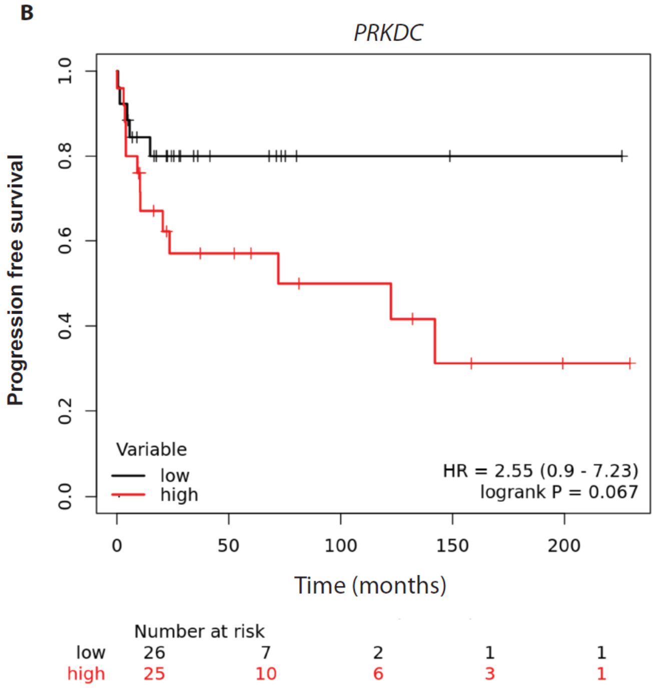

Figure S5. (**A–B**) Expression of *TP53BP1* (**A**) and *PRKDC* (**B**) in patients with a different degree of progression free-survival.

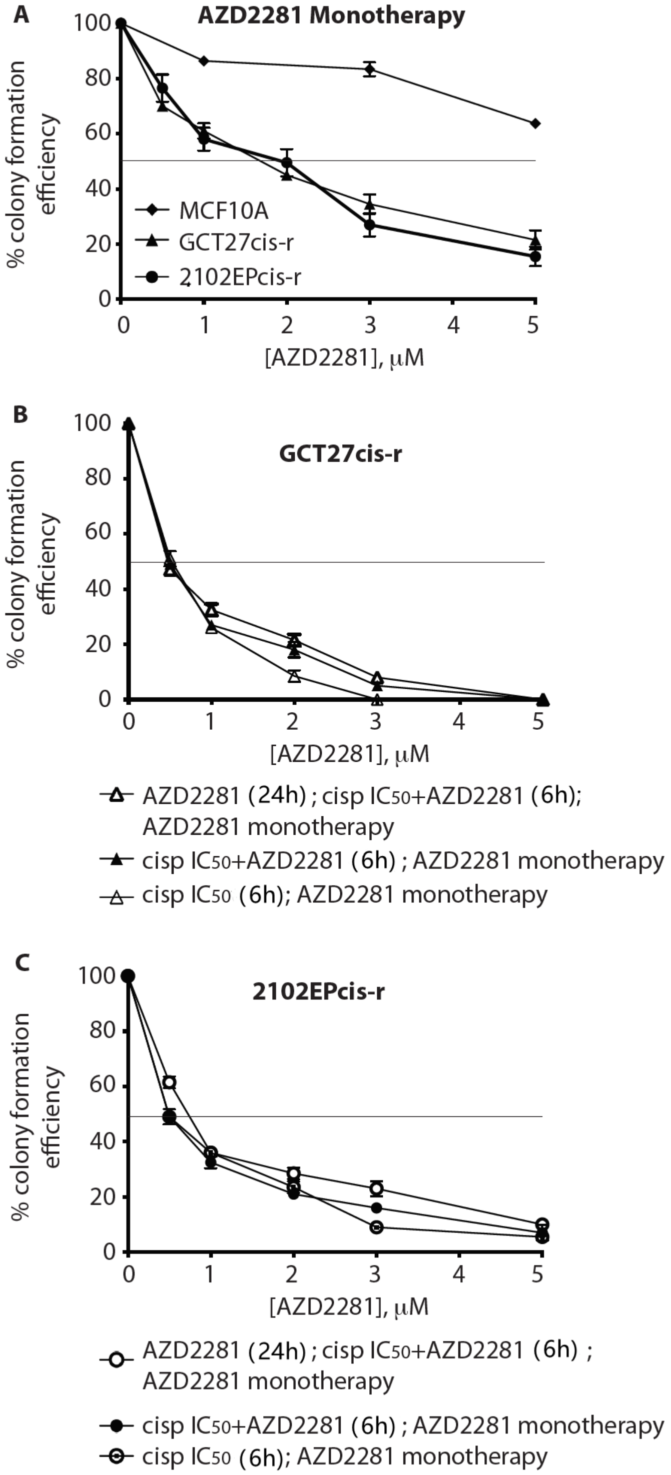

Figure S6. Dose-response survival curves of MCF10A and EC cell lines upon treatment with AZD2281 or AZD2281/cisplatin combined therapy. (**A**) Dose-response survival curves of the indicated cell lines following treatment with the PARPi AZD2281 monotherapy, at the doses indicated. Survival was assessed by colony assay. The dotted line intercepts the half maximal inhibitory concentration (IC_{50}). (**B**–**C**) Colony assay of the indicated cis-r cell lines exposed to three distinct cisplatin/AZD2281 combined treatments. *AZD2281 (24 h); cisp IC_{50}+AZD2281 (6 h); AZD2281 monotherapy*: cells were pretreated with AZD2281 for 24 h. After a wash out were exposed to cisplatin (IC_{50}) in combination with increasing doses of AZD2281 for 6 h. At the end of the incubation time cells were cultured with AZD2281 as single agent. *Cisp IC_{50}+ AZD2281 (6 h); AZD2281 monotherapy*: cells were treated with the IC_{50} dose of cisplatin in combination with increasing doses of AZD2281 for 6 h. After a washout, cells were incubated with the indicated concentrations of PARPi. *Cisp IC_{50} (6 h) + PARPi monotherapy*: cells were treated with the IC_{50} dose of cisplatin for 6 h. After a washout, were incubated with the indicated increasing concentrations of PARPi alone. Data are mean value ± s.d. of two independent experiments, each done in triplicate. The dotted line intercepts the half maximal inhibitory concentration (IC_{50}).

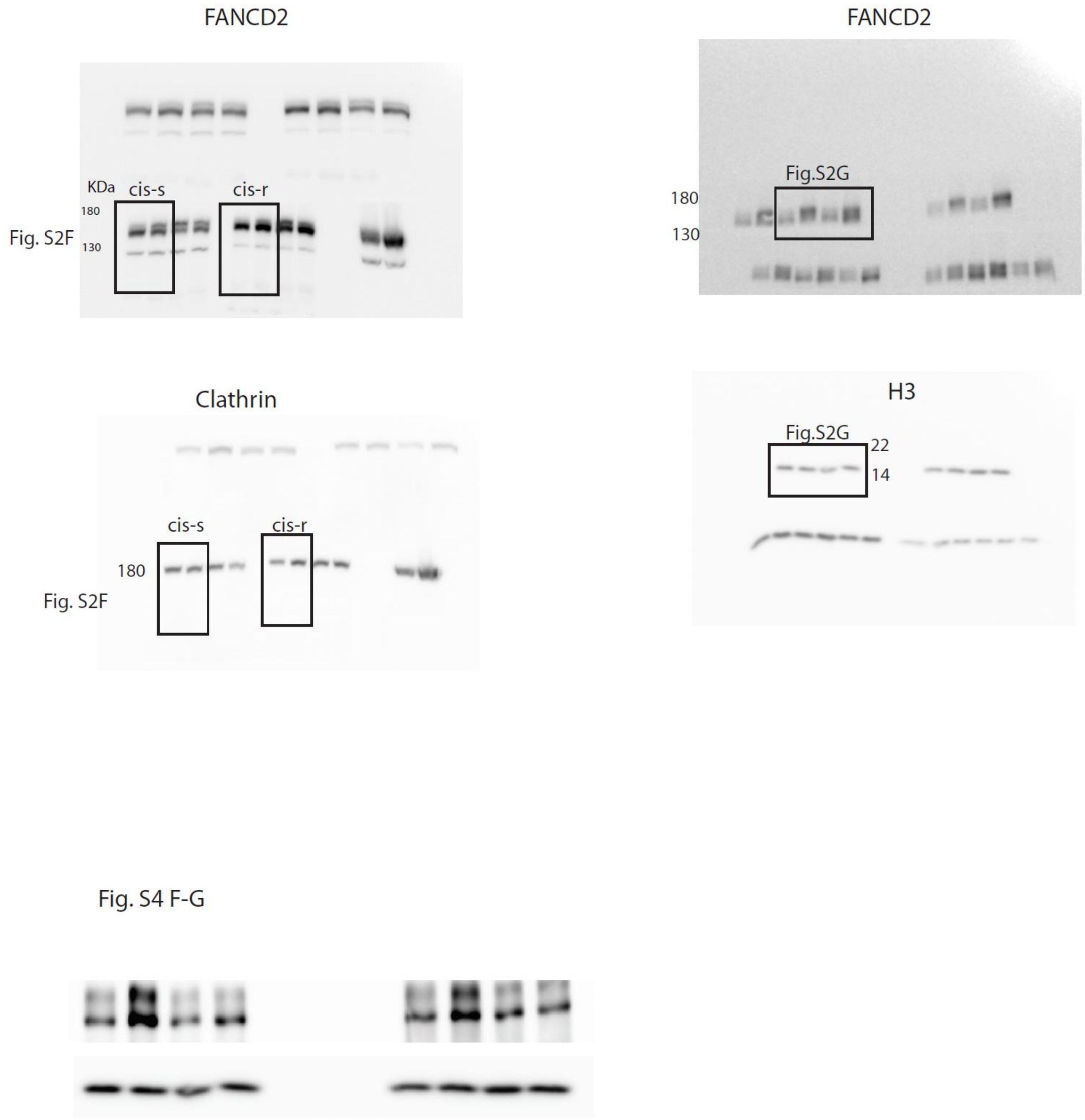

Figure S7. Original blots of Figures S2,S4.

Table S1. Combination Index (CI) of cisplatin and DNAPKcs inhibitor (DNAPKi) co-treatments, in the indicated cell lines.

6h cisp 3µM + 72h DNAPKi	[NU7441]	GCT27cis-s	2102EPcis-s
		CI	CI
	1µM	1.8	1.98
	2.5µM	1.9	1.92
	5µM	2.4	1.7
	[NU-7441]	GCT27cis-r	2101EPcis-r
		CI	CI
	1µM	2.7	2
	2.5µM	2.7	N.D.
	5µM	N.D.	N.D.
